# Supplementary material for: A meta-analytic review and conceptual model of the antecedents and outcomes of goal adjustment in response to striving difficulties
Source: Nat Hum Behav. 2025 Nov 13;10(2):317–32. doi: 10.1038/s41562-025-02312-4 (PMC12932095; doi:10.1038/s41562-025-02312-4)
Supplement: Supplementary file 1 — Supplementary moderator analysis. [file 41562_2025_2312_MOESM1_ESM.pdf]

nature human behaviour

Supplementary information https://doi.org/10.1038/s41562-025-02312-4

A meta-analytic review and conceptual model of the antecedents and outcomes of goal adjustment in response to striving difficulties

In the format provided by the authors and unedited

Contents

Moderator Analysis Summary (SI-1) 2
- Antecedents of Goal Disengagement 2
- Antecedents of Goal Reengagement 5
- Antecedents of Goal Striving Flexibility 12
- Goal Disengagement Outcome Variables 17
- Goal Reengagement Outcome Variables 22
- Goal Striving Flexibility Outcome Variables 29

Search Terms (SI-2) 37

Additional References Included in Meta-Analysis (SI-3) 38

Moderator Analysis Summary (SI-1)

Antecedents of Goal Disengagement

We examined moderators of goal inhibiting variables. Study quality emerged as a significant moderator, with higher quality studies associated with lower effect sizes. Likewise, study design explained substantial heterogeneity in the model, although no individual study design category differed significantly from others. Country of study was also a significant moderator overall. Effect sizes were significantly smaller in several countries, including Canada, China, England, Germany, Israel, Japan, Netherlands, Pakistan, UK, and USA. In contrast, studies coded as having an unspecified country had slightly larger effects. The population sampled also significantly moderated effect sizes. Specifically, studies of the general population reported significantly lower effect sizes, whereas studies of online samples and students evinced significantly larger effects. Finally, when we included all significant moderators in a joint model, the overall test of moderators was not statistically significant, likely due to multicollinearity or overlapping variance across predictors.

SI1.1 Goal inhibiting variables

Moderator	Level	β Estimate	95% CI	p-value	Overall F-test (df)	Overall p-value
**Age**	(continuous)	0.0002	[-0.0027, 0.0032]	0.8702	F(1, 56) = 0.03	0.8702
**Study Quality**	(continuous or dichotomous)	-0.8534	[-1.0866, -0.6202]	<.0001 ***	F(1, 56) = 53.74	<.0001
**Study Design**	Lab-Based	0.0287	[-0.1828, 0.2401]	0.7868		
	Longitudinal	-0.0327	[-0.2396, 0.1742]	0.7527		
	Observational	-0.1469	[-0.3523, 0.0585]	0.1573	F(3, 54) = 15.33	<.0001
**Country**	Canada	-0.6415	[-0.8375, -0.4455]	<.0001 ***		
	China	-0.2272	[-0.3347, -0.1197]	0.0001 ***		
	England	-0.5192	[-0.6875, -0.3508]	<.0001 ***		
	Georgia	-0.0981	[-0.2570, 0.0607]	0.2196		
	Germany	-0.2052	[-0.3970, -0.0134]	0.0366 *		

Moderator	Level	β Estimate	95% CI	p-value	Overall F-test (df)	Overall p-value
	Israel	-0.5497	[-0.7291, -0.3704]	<.0001 ***		
	Japan	-0.2622	[-0.3756, -0.1489]	<.0001 ***		
	Multiple	-0.2878	[-0.3945, -0.1812]	<.0001 ***		
	Netherlands	-0.3524	[-0.5581, -0.1468]	0.0012 **		
	Pakistan	-0.6143	[-0.7430, -0.4856]	<.0001 ***		
	Switzerland	-0.1291	[-0.3043, 0.0460]	0.1444		
	UK	-0.3478	[-0.4590, -0.2365]	<.0001 ***		
	Unspecified	0.1659	[0.0256, 0.3062]	0.0216 *		
	USA	-0.3366	[-0.4486, -0.2246]	<.0001 ***	F(14, 43) = 20.25	<.0001
**Population**	Business Owners / Managers	-0.0219	[-0.1329, 0.0892]	0.6942		
	General Population	-0.2700	[-0.4014, -0.1386]	0.0001 ***		
	Medical Condition	-0.1518	[-0.3083, 0.0047]	0.057 .		
	Online	0.1816	[0.0819, 0.2812]	0.0006 ***		
	Parents	0.2081	[-0.0166, 0.4327]	0.0688 .		
	Student	0.1772	[0.0680, 0.2864]	0.0020 **	F(6, 51) = 23.53	<.0001
**All Significant Moderators Combined**	(Intercept only shown)	—	—	—	F(22, 35) = 1.25	0.2705
	Country: Canada	-1.5205	[-2.8491, -0.1920]	0.0261 *		
	Country: Pakistan	-1.5421	[-2.6451, -0.4391]	0.0075 **		
	Country: USA	-0.5763	[-1.1645, 0.0118]	0.0545 .		
	Population: General Population	1.0844	[0.0463, 2.1225]	0.0411 *		

The moderator analyses of the goal enhancing variables category identified several significant predictors of variation in effect sizes. Age significantly moderated the results, with older age associated with more positive effects. Study quality also significantly influenced effect sizes, with higher study quality associated with more positive effects. Study design had a significant overall moderation effect, although none of the individual study types differed significantly. Country was a strong moderator, with studies from Georgia, Germany, Italy, UK, USA, and unspecified countries reporting significantly more positive effect sizes, but studies from Japan and Pakistan reporting significantly more negative effects. In addition, population type significantly moderated the results, with studies involving the general population, online samples, parents, students, and young adults/adolescents showing significantly more positive effects.

SI1.2 Goal enhancing variables

Moderator Type	Term	Estimate	SE	t	p	95% CI (LB)	95% CI (UB)	Sig
**Age**	Intercept	-0.1342	0.0448	-2.998	.0039	-0.2237	-0.0448	**
	Age (z)	0.0026	0.0011	2.291	.0253	0.0003	0.0048	*
**Study Quality**	Intercept	-0.1289	0.0440	-2.928	.0047	-0.2168	-0.0409	**
	Study Quality	0.3856	0.1268	3.042	.0034	0.1323	0.6389	**
**Study Design**	Intercept	-0.0414	0.0841	-0.493	.6240	-0.2096	0.1268	
	Lab-Based	-0.1097	0.0755	-1.452	.1516	-0.2608	0.0414	
	Longitudinal	-0.0414	0.0724	-0.572	.5696	-0.1861	0.1033	
	Observational	-0.1153	0.0726	-1.588	.1174	-0.2606	0.0299	
**Country**	Intercept	-0.1694	0.0526	-3.218	.0022	-0.2750	-0.0638	**
	England	-0.0933	0.0831	-1.122	.2668	-0.2602	0.0735	
	Georgia	0.3495	0.0560	6.244	<.0001	0.2372	0.4618	***
	Germany	0.2456	0.0489	5.026	<.0001	0.1475	0.3436	***
	Italy	0.1078	0.0536	2.010	.0496	0.0002	0.2154	*
	Japan	-0.1855	0.0446	-4.162	.0001	-0.2749	-0.0961	***

Moderator Type	Term	Estimate	SE	t	p	95% CI (LB)	95% CI (UB)	Sig
	Lithuania	-0.0556	0.0804	-0.692	.4919	-0.2169	0.1056	
	Multiple	-0.0584	0.0482	-1.212	.2309	-0.1550	0.0383	
	Pakistan	-0.4228	0.0465	-9.099	<.0001	-0.5161	-0.3296	***
	Switzerland	-0.0766	0.0601	-1.275	.2080	-0.1971	0.0440	
	UK	0.1301	0.0416	3.131	.0029	0.0467	0.2135	**
	Unspecified	0.1764	0.0411	4.289	<.0001	0.0939	0.2589	***
	USA	0.2099	0.0352	5.967	<.0001	0.1393	0.2805	***
**Population**	Intercept	-0.3227	0.0705	-4.579	<.0001	-0.4638	-0.1816	***
	Business Owners/Managers	-0.0138	0.0551	-0.251	.8027	-0.1242	0.0965	
	General Population	0.2555	0.0577	4.428	<.0001	0.1400	0.3710	***
	Online	0.1889	0.0510	3.708	.0005	0.0869	0.2909	***
	Parents	0.2655	0.0870	3.050	.0034	0.0912	0.4397	**
	Students	0.2937	0.0524	5.608	<.0001	0.1889	0.3985	***
	Young Adults/Adolescents	0.2073	0.0600	3.455	.0010	0.0872	0.3275	**

Antecedents of Goal Reengagement

We examined moderators of the association between affect enhancing variables and goal reengagement. Age, study quality, sample country, and sample population were significant. Older participants and participants sampled from the Spain or USA exhibited larger effect sizes, whereas studies rated as higher quality or testing university students as participants had smaller effect sizes. In combination, these moderators did not account for a substantive proportion of between-study variation in the association between affect enhancing variables and goal reengagement.

SI1.3 Affect enhancing variables

Moderator Type	Term	Estimate	SE	t	df	95% CI (LB)	95% CI (UB)	Sig
**Age**	Intercept	0.3178	0.0390	8.14	12	0.2328	0.4028	***
	Age (z)	0.0213	0.0015	14.26	12	0.0180	0.0245	***
**Study Quality**	Intercept	0.2468	0.0769	3.21	12	0.0792	0.4144	**

Moderator Type	Term	Estimate	SE	t	df	95% CI (LB)	95% CI (UB)	Sig
	Study Quality (z)	-1.7707	0.3448	-5.14	12	-2.5219	-1.0195	***
**Study Design**	Intercept	0.2622	0.0853	3.07	12	0.0762	0.4481	**
	Observational	-0.0022	0.0629	-0.03	12	-0.1392	0.1349	
**Country**	Intercept	0.2053	0.0508	4.04	7	0.0853	0.3254	**
	Germany	-0.0130	0.1039	-0.13	7	-0.2586	0.2326	
	Japan	-0.1592	0.0677	-2.35	7	-0.3193	0.0009	.
	Lithuania	-0.0523	0.0995	-0.53	7	-0.2876	0.1830	
	Spain	0.6029	0.0589	10.23	7	0.4636	0.7423	***
	Unspecified	0.1176	0.1091	1.08	7	-0.1405	0.3757	
	USA	0.4870	0.1440	3.38	7	0.1465	0.8274	*
**Population**	Intercept	0.6364	0.0501	12.70	12	0.5272	0.7456	***
	Student	-0.4995	0.0401	-12.47	12	-0.5868	-0.4122	***
**All Significant Moderators Combined**	Intercept	0.3065	0.1887	1.62	4	-0.2173	0.8303	
	Age (z)	0.0567	0.0733	0.77	4	-0.1470	0.2603	
	Study Quality (z)	-0.1380	0.9172	-0.15	4	-2.6847	2.4086	
	Germany	-0.3703	0.4512	-0.82	4	-1.6230	0.8824	
	Japan	-0.1975	0.1379	-1.43	4	-0.5804	0.1853	
	Lithuania	0.0085	0.1636	0.05	4	-0.4457	0.4627	
	Spain	-0.7439	1.7029	-0.44	4	-5.4718	3.9841	
	Unspecified	-0.0758	0.2799	-0.27	4	-0.8529	0.7014	
	USA	0.0095	0.5997	0.02	4	-1.6556	1.6747	
	Student	0.4666	0.6882	0.68	4	-1.4441	2.3773	

Moderator analysis of adaptive personality traits indicated that age, study quality, study design, sample country, and sample population all influenced effect sizes, with older participants, higher quality studies, lab-based studies, studies sampling participants from Australia, Korea, or the UK, and studies sampling business owners/managers or parents producing larger effect sizes.

SI1.4 Adaptive personality traits

Moderator Type	Term	Estimate	SE	t	df	95% CI (LB)	95% CI (UB)	Sig
**Age**	Intercept	0.2193	0.0339	6.47	35	0.1505	0.2881	***
	Age	0.0065	0.0010	6.41	35	0.0045	0.0086	***
**Study Quality**	Intercept	0.1970	0.0288	6.85	35	0.1386	0.2555	***
	Study Quality	0.1285	0.1363	0.94	35	-0.1482	0.4052	
**Study Design**	Intercept	0.3588	0.0721	4.97	34	0.2122	0.5054	***
	Longitudinal	-0.1283	0.0785	-1.64	34	-0.2877	0.0311	
	Observational	-0.1737	0.0665	-2.61	34	-0.3089	-0.0385	*
**Country**	Intercept	0.6027	0.0799	7.54	25	0.4381	0.7673	***
	China	-0.7768	0.0932	-8.33	25	-0.9687	-0.5848	***
	Germany	-0.4887	0.0719	-6.80	25	-0.6368	-0.3406	***
	Israel	-0.5188	0.1214	-4.27	25	-0.7689	-0.2687	***
	Japan	-0.3140	0.0692	-4.54	25	-0.4566	-0.1715	***
	Korea	-0.2588	0.1223	-2.12	25	-0.5106	-0.0070	*
	Lithuania	-0.4118	0.0800	-5.15	25	-0.5765	-0.2470	***
	Netherlands	-0.3880	0.1337	-2.90	25	-0.6633	-0.1126	**
	Slovakia	-0.3040	0.0709	-4.29	25	-0.4500	-0.1579	***
	UK	-0.3716	0.1110	-3.35	25	-0.6001	-0.1431	**
	Unspecified	-0.3200	0.0815	-3.92	25	-0.4879	-0.1520	***
	USA	-0.2610	0.0771	-3.39	25	-0.4197	-0.1023	**
**Population**	Intercept	0.5753	0.0681	8.45	30	0.4363	0.7144	***
	General Population	-0.3587	0.0601	-5.96	30	-0.4815	-0.2358	***
	Medical Condition	-0.4140	0.0826	-5.01	30	-0.5826	-0.2454	***
	Online	-0.4688	0.0600	-7.81	30	-0.5913	-0.3462	***
	Parents	-0.4011	0.1564	-2.56	30	-0.7206	-0.0816	*

Moderator Type	Term	Estimate	SE	t	df	95% CI (LB)	95% CI (UB)	Sig
	Students	-0.3118	0.0582	-5.36	30	-0.4307	-0.1930	***
	Young Adult/Adolescent	-0.5395	0.0635	-8.49	30	-0.6692	-0.4098	***
**All Moderators**	Intercept	-4.2044	6.4021	-0.66	16	-17.7763	9.3675	
	Age	0.2081	0.2457	0.85	16	-0.3128	0.7289	
	Longitudinal	-3.0328	3.5208	-0.86	16	-10.4965	4.431	
	Observational	-2.3894	2.7412	-0.87	16	-8.2004	3.4216	
	China	-0.6992	0.5577	-1.25	16	-1.8814	0.4831	
	Germany	-1.159	1.0976	-1.06	16	-3.4859	1.1679	
	Israel	-7.7502	8.9049	-0.87	16	-26.6278	11.1274	
	Japan	-0.2331	0.506	-0.46	16	-1.3057	0.8395	
	Korea	-3.9418	4.4203	-0.89	16	-13.3124	5.4288	
	Lithuania	-0.4251	0.5109	-0.83	16	-1.5081	0.658	
	Netherlands	-8.3225	9.6804	-0.86	16	-28.844	12.1989	
	Slovakia	-2.1986	2.7226	-0.81	16	-7.9702	3.573	
	UK	-5.2354	6.332	-0.83	16	-18.6587	8.1879	
	Unspecified	-5.465	6.5445	-0.84	16	-19.3387	8.4087	
	USA	-1.411	0.976	-1.45	16	-3.4801	0.658	
	General Population	9.7247	12.5312	0.78	16	-16.8402	36.2896	
	Medical Condition	11.287	14.3454	0.79	16	-19.1238	41.6978	
	Online	6.9181	9.2483	0.75	16	-12.6873	26.5236	
	Parents	5.4541	6.5652	0.83	16	-8.4636	19.3717	
	Students	8.4971	10.7488	0.79	16	-14.2894	31.2836	
	Young Adults / Adolescents	10.7741	13.5437	0.8	16	-17.9372	39.4853	

Moderator analyses revealed that both age and population type significantly influenced effect sizes for the association between developmental/contextual enablers and goal reengagement. Studies with older participants reported stronger effects. The effect of population type also varied meaningfully: studies with young adult or adolescent samples showed smaller effects, and there was some indication that samples involving medical conditions yielded weaker effects as well. No other moderators—including study design, country, or risk of bias—substantively accounted for differences in effect sizes. When we entered all significant moderators into a combined model, none remained statistically significant.

SI1.5 Developmental/contextual enablers

Moderator Type	Term	Estimate	SE	t	df	p	95% CI (LB)	95% CI (UB)
**Age**	Intercept	0.2459	0.0175	14.02	9	<.001	0.2062	0.2855
	Age	0.0039	0.0007	5.81	9	.0003	0.0024	0.0054
**Study quality**	Intercept	0.1832	0.0424	4.32	9	.0019	0.0873	0.2792
	Higher quality	-0.1819	0.3263	-0.56	9	.591	-0.9200	0.5562
**Study Design**	Intercept	0.1165	0.0661	1.76	8	.116	-0.0359	0.2690
	Observational	0.0973	0.0487	2.00	8	.081	-0.0150	0.2097
	RCT	-0.1144	0.0807	-1.42	8	.194	-0.3005	0.0717
**Country**	Intercept	0.0824	0.0756	1.09	7	.312	-0.0963	0.2611
	Germany	0.1365	0.0662	2.06	7	.078	-0.0199	0.2930
	Netherlands	0.0307	0.0968	0.32	7	.761	-0.1982	0.2596
	USA	0.0934	0.0617	1.51	7	.174	-0.0524	0.2392
**Population**	Intercept	0.3153	0.0363	8.69	6	.0001	0.2265	0.4041
	General Population	0.0053	0.0641	0.08	6	.937	-0.1516	0.1621
	Medical Condition	-0.1505	0.0636	-2.37	6	.056	-0.3063	0.0052
	Online	0.0965	0.0620	1.56	6	.171	-0.0552	0.2482
	Young Adult / Adolescent	-0.1980	0.0441	-4.49	6	.0041	-0.3059	-0.0901
**All Significant Moderators**	Intercept	-0.2639	0.6373	-0.41	5	.696	-1.9021	1.3743
	Age (z)	0.0239	0.0261	0.92	5	.401	-0.0431	0.0909
	General Population	0.0692	0.2074	0.33	5	.752	-0.4640	0.6023
	Medical Condition	0.4305	0.6936	0.62	5	.562	-1.3525	2.2136
	Online	0.4367	0.4157	1.05	5	.342	-0.6320	1.5053
	Young Adult / Adolescent	1.1676	1.4968	0.78	5	.471	-2.6800	5.0152

Age, study design, and country all significantly moderated the association between agency enhancing variables and goal reengagement. Cross-sectional studies yielded the largest effects, with both longitudinal and observational designs producing notably smaller estimates. Studies conducted in Israel of USA, or with unspecified country information, evinced larger effects. In a combined model including all significant moderators, study design

remained a significant predictor of variation in effects, whereas age and country did not retain their individual contributions.

SI1.6 Agency enhancing variables

Moderator Type	Term	Estimate	SE	t	df	p	95% CI (LB)	95% CI (UB)
**Age**	Intercept	0.1774	0.0235	7.57	12	<.001	0.1263	0.2285
	Age (z)	0.0154	0.0021	7.27	12	<.001	0.0108	0.0200
**Study quality**	Intercept	0.1389	0.0259	5.36	12	.0002	0.0825	0.1953
	Higher quality	-0.1559	0.2040	-0.76	12	.4595	-0.6005	0.2886
**Study Design**	Intercept (Cross-sectional)	0.5919	0.0943	6.28	11	<.001	0.3844	0.7994
	Longitudinal	-0.4088	0.0894	-4.57	11	.0008	-0.6055	-0.2120
	Observational	-0.5965	0.0776	-7.68	11	<.001	-0.7674	-0.4256
**Country**	Intercept (Ref = non-listed)	-0.0100	0.0455	-0.22	10	.8302	-0.1115	0.0915
	Israel	0.2548	0.1108	2.30	10	.0443	0.0079	0.5017
	Unspecified	0.6736	0.0860	7.83	10	<.001	0.4820	0.8652
	USA	0.1302	0.0493	2.64	10	.0247	0.0203	0.2401
**Population**	Intercept	0.2448	0.1102	2.22	11	.0483	0.0022	0.4874
	Online	0.2305	0.1216	1.90	11	.0844	-0.0370	0.4981
	Student	-0.1561	0.1128	-1.38	11	.1938	-0.4043	0.0921
**All Significant Moderators**	Intercept	0.5434	0.1152	4.72	8	.0015	0.2777	0.8091
	Age (z)	0.0086	0.0048	1.80	8	.1101	-0.0024	0.0196
	Longitudinal	-0.4872	0.1622	-3.00	8	.0170	-0.8612	-0.1131
	Observational	-0.4846	0.1594	-3.04	8	.0161	-0.8523	-0.1170
	Country: Israel	0.0667	0.1800	0.37	8	.7204	-0.3484	0.4819
	Country: USA	0.0906	0.1013	0.89	8	.3974	-0.1430	0.3242

Moderator analyses for the association between reengagement and goal inhibiting variables identified several significant predictors of effect size variation across studies. Age significantly moderated effects, with older participant samples showing weaker effects. Study quality was a strong moderator: studies with higher quality showed substantially smaller

effects. Country of study was a strong and consistent moderator; effect sizes were significantly lower in nearly all countries compared to the reference group. Population type also moderated effects: effects were stronger in students and online samples, and significantly weaker in medical populations. In a combined model, country remained the most robust moderator, with the effect size significantly lower in countries like Israel, Netherlands, UK, and USA, whereas other moderators (i.e., age, risk of bias, population, study design) no longer contributed significantly to the model.

SI1.7 Goal inhibiting variables

Moderator Type	Term	Estimate	SE	t	df	P	95% CI (LB)	95% CI (UB)
**Age**	Intercept	0.1883	0.0578	3.26	33	.0026	0.0708	0.3059
	Age (z)	-0.0143	0.0020	-7.09	33	<.0001	-0.0184	-0.0102
**Risk of Bias**	Intercept	0.1276	0.0425	3.00	33	.0051	0.0411	0.2141
	Higher ROB	-1.7594	0.1098	-16.02	33	<.0001	-1.9829	-1.5360
**Study Design**	Intercept (Ref = cross-sectional)	0.0947	0.1365	0.69	30	.4933	-0.1841	0.3735
	Lab-based	0.0664	0.1152	0.58	30	.5686	-0.1689	0.3017
	Longitudinal	-0.1002	0.1055	-0.95	30	.3497	-0.3157	0.1153
	Observational	0.0155	0.1035	0.15	30	.8818	-0.1959	0.2269
	RCT	-0.0975	0.1616	-0.60	30	.5508	-0.4277	0.2326
**Country**	Intercept	0.4921	0.0547	9.00	22	<.0001	0.3788	0.6055
	Canada	-0.4642	0.0857	-5.42	22	<.0001	-0.6418	-0.2865
	China	-0.3456	0.0335	-10.32	22	<.0001	-0.4151	-0.2762
	England	-0.5326	0.0832	-6.40	22	<.0001	-0.7050	-0.3601
	Germany	-0.5208	0.1291	-4.03	22	.0006	-0.7885	-0.2531
	Israel	-0.7872	0.0761	-10.34	22	<.0001	-0.9452	-0.6293
	Japan	-0.5291	0.0584	-9.06	22	<.0001	-0.6502	-0.4080
	Multiple	-0.4241	0.0606	-7.00	22	<.0001	-0.5498	-0.2985
	Netherlands	-0.7534	0.0849	-8.88	22	<.0001	-0.9293	-0.5774
	Spain	-0.6450	0.0545	-11.84	22	<.0001	-0.7579	-0.5320
	UK	-0.4756	0.0594	-8.00	22	<.0001	-0.5988	-0.3524
	Unspecified	-0.2424	0.0747	-3.25	22	.0037	-0.3972	-0.0875
	USA	-0.4232	0.0701	-6.03	22	<.0001	-0.5687	-0.2778
**Population**	Intercept	0.0146	0.0394	0.37	29	.7132	-0.0660	0.0952
	General Pop	0.0752	0.0803	0.94	29	.3566	-0.0890	0.2394

Moderator Type	Term	Estimate	SE	t	df	P	95% CI (LB)	95% CI (UB)
	Medical Cond.	-0.2105	0.0648	-3.25	29	.0029	-0.3430	-0.0780
	Online	0.1195	0.0489	2.44	29	.0210	0.0194	0.2195
	Parents	0.1732	0.1059	1.64	29	.1129	-0.0435	0.3898
	Student	0.3715	0.0413	9.00	29	<.0001	0.2871	0.4559
**All Significant Moderators**	Intercept	1.7469	1.3516	1.29	17	.2135	-1.1046	4.5985
	Age (z)	0.0263	0.0554	0.47	17	.6412	-0.0906	0.1431
	Risk of Bias (z)	1.2346	2.4070	0.51	17	.6146	-3.8438	6.3130
	Lab-Based	-0.0854	0.5921	-0.14	17	.8869	-1.3346	1.1637
	Longitudinal	-0.7879	1.3905	-0.57	17	.5784	-3.7216	2.1459
	Observational	-0.7434	1.4065	-0.53	17	.6040	-3.7108	2.2241
	RCT	-1.8107	1.5977	-1.13	17	.2728	-5.1815	1.5602
	Canada	-0.8728	0.2346	-3.72	17	.0017	-1.3678	-0.3779
	Israel	-1.7552	0.4852	-3.62	17	.0021	-2.7789	-0.7314
	Netherlands	-1.7994	0.7567	-2.38	17	.0294	-3.3958	-0.2029
	UK	-1.5208	0.5533	-2.75	17	.0137	-2.6882	-0.3534
	USA	-0.8276	0.2902	-2.85	17	.0110	-1.4398	-0.2154

Antecedents of Goal Striving Flexibility

Moderator analyses for the association between affect enhancing variables and goal striving flexibility revealed that study design significantly moderated effect sizes, with observational designs associated with stronger effects. Country of study was also a significant moderator: effects were significantly stronger in Spain and the Netherlands. Overall, the model combining all significant moderators confirmed the Netherlands as a consistent and strong positive moderator of effect size.

SI1.8 affect enhancing variables

Moderator Type	Term	Estimate	SE	t	df	p	95% CI (LB)	95% CI (UB)
**Age**	Intercept	0.5952	0.0380	15.68	6	<.0001	0.5023	0.6881
	Age (z)	-0.0031	0.0029	-1.06	6	.3309	-0.0102	0.0041
**Study Quality**	Intercept	0.5876	0.0320	18.39	6	<.0001	0.5094	0.6658
	Higher quality	0.0194	0.0999	0.19	6	.8527	-0.2250	0.2637
**Study Design**	Intercept (Ref = non-observ.)	0.4749	0.0536	8.86	6	.0001	0.3438	0.6060

Moderator Type	Term	Estimate	SE	t	df	p	95% CI (LB)	95% CI (UB)
	Observational	0.1814	0.0458	3.96	6	.0075	0.0692	0.2935
**Country**	Intercept	0.5161	0.0295	17.51	4	<.0001	0.4343	0.5980
	Netherlands	0.3514	0.0697	5.04	4	.0073	0.1580	0.5447
	Spain	0.1537	0.0474	3.24	4	.0317	0.0220	0.2854
	Unspecified	-0.0326	0.0577	-0.56	4	.6029	-0.1928	0.1277
**Population**	Intercept	0.5783	0.0265	21.86	5	<.0001	0.5103	0.6463
	Medical Condition	0.0912	0.0456	2.00	5	.1022	-0.0261	0.2085
	Unspecified	-0.0948	0.0562	-1.68	5	.1528	-0.2393	0.0498
**All Significant Moderators**	Intercept	0.5167	0.0397	13.01	4	.0002	0.4064	0.6270
	Observational Design	-0.0332	0.0691	-0.48	4	.6561	-0.2249	0.1586
	Netherlands	0.3838	0.0929	4.13	4	.0145	0.1258	0.6418
	Spain	0.1857	0.0712	2.61	4	.0594	-0.0118	0.3833

In the moderator analysis considering adaptive personality traits and goal striving flexibility, age emerged as a robust negative moderator, with older samples associated with smaller effect sizes. Study design was also a significant moderator, with observational studies showing stronger effects. Moreover, country of study moderated effects: we observed larger effect sizes in studies from Germany, Iran, USA, and those from unspecified countries. Population type significantly moderated effects as well; unspecified populations were associated with stronger effects, whereas general population samples were marginally weaker. However, when we entered all significant moderators into a joint model, none remained statistically significant.

SI1.9 adaptive personality traits

Moderator Type	Term	Estimate	SE	t	df	p	95% CI (LB)	95% CI (UB)
**Age**	Intercept	0.2319	0.0287	8.08	15	<.0001	0.1707	0.2930
	Age (z)	-0.0087	0.0011	-7.62	15	<.0001	-0.0112	-0.0063
**Study Quality**	Intercept	0.2284	0.0378	6.05	15	<.0001	0.1479	0.3089
	Higher quality	-0.0048	0.0737	-0.07	15	.9490	-0.1618	0.1522
**Study Design**	Intercept (Ref = non-observ.)	0.1455	0.0411	3.54	15	.0030	0.0580	0.2330

Moderator Type	Term	Estimate	SE	t	df	p	95% CI (LB)	95% CI (UB)
	Observational	0.1139	0.0262	4.34	15	.0006	0.0580	0.1697
**Country**	Intercept	0.0666	0.0354	1.88	12	.0845	-0.0105	0.1438
	Germany	0.4237	0.0519	8.16	12	<.0001	0.3107	0.5368
	Iran	0.1501	0.0463	3.24	12	.0071	0.0492	0.2510
	USA	0.1820	0.0289	6.30	12	<.0001	0.1191	0.2450
	Unspecified	0.4727	0.0770	6.14	12	<.0001	0.3051	0.6404
**Population**	Intercept	0.3315	0.0689	4.81	13	.0003	0.1827	0.4802
	General Population	-0.1182	0.0569	-2.08	13	.0581	-0.2411	0.0047
	Medical Condition	-0.0956	0.0681	-1.40	13	.1837	-0.2428	0.0515
	Unspecified	0.2499	0.0941	2.65	13	.0198	0.0465	0.4532
**All Significant Moderators**	Intercept	0.0980	0.2267	0.43	11	.6739	-0.4010	0.5970
	Age	-0.0029	0.0115	-0.25	11	.8050	-0.0283	0.0225
	Observational Design	0.1347	0.2462	0.55	11	.5951	-0.4071	0.6766
	Germany	0.4010	0.3229	1.24	11	.2402	-0.3098	1.1117
	Iran	-0.0585	0.1726	-0.34	11	.7411	-0.4385	0.3215
	Unspecified	0.2224	0.3006	0.74	11	.4749	-0.4393	0.8841

Moderator analysis for the developmental/contextual enablers category showed that age was a significant positive moderator: older samples were associated with slightly larger effect sizes. Study quality also significantly moderated effects, with higher quality studies showing significantly smaller effect sizes. Study design emerged as a significant moderator, with observational designs associated with smaller effects. In addition, country moderated effects: studies conducted in USA and unspecified countries reported larger effects. Population type significantly moderated effects as well: student and medical samples evinced smaller effects, whereas effects were larger for unspecified populations. However, when we included all significant moderators in a single model, none remained statistically significant.

SI1.10 development/contextual enablers

Moderator Type	Term	Estimate	SE	t	df	p	95% CI (LB)	95% CI (UB)
**Age**	Intercept	0.1041	0.0188	5.55	29	<.0001	0.0657	0.1425
	zAge	0.0014	0.0004	3.25	29	.0029	0.0005	0.0023
**Study quality**	Intercept	0.0929	0.0151	6.15	29	<.0001	0.0620	0.1238
	Higher quality	-0.3130	0.0836	-3.75	29	.0008	-0.4839	-0.1421
**Study Design**	Intercept	0.1506	0.0394	3.82	29	.0006	0.0700	0.2312
	Observational	-0.1110	0.0233	-4.76	29	<.0001	-0.1587	-0.0633
**Country**	Intercept	0.0301	0.0287	1.05	28	.3035	-0.0287	0.0889
	USA	0.1088	0.0211	5.15	28	<.0001	0.0656	0.1521
	Unspecified	0.1011	0.0316	3.19	28	.0035	0.0362	0.1659
**Population**	Intercept	0.2283	0.0505	4.52	25	.0001	0.1242	0.3323
	General Population	-0.0811	0.0546	-1.49	25	.1500	-0.1935	0.0314
	Medical Condition	-0.1432	0.0674	-2.13	25	.0436	-0.2821	-0.0044
	Online Sample	0.0617	0.0659	0.94	25	.3584	-0.0741	0.1975
	Student Sample	-0.1658	0.0504	-3.29	25	.0030	-0.2697	-0.0620
	Unspecified	0.1258	0.0874	1.44	25	.1623	-0.0541	0.3058
**All Moderators**	Intercept	0.5774	0.5076	1.14	20	.2688	-0.4814	1.6362
	Age	-0.0017	0.0085	-0.20	20	.8450	-0.0195	0.0161
	Study quality	-0.8505	1.0411	-0.82	20	.4236	-3.0221	1.3212
	Observational Design	-0.1804	0.1998	-0.90	20	.3771	-0.5971	0.2363
	USA	0.0122	0.1888	0.06	20	.9493	-0.3818	0.4061
	Unspecified Country	-0.0031	0.3083	-0.01	20	.9921	-0.6462	0.6401
	GenPop	-0.2818	0.3097	-0.91	20	.3737	-0.9277	0.3642
	Medical Condition	-0.4787	0.5284	-0.91	20	.3758	-1.5808	0.6235
	Online	-0.2486	0.4078	-0.61	20	.5490	-1.0992	0.6021
	Student	-0.3776	0.4695	-0.80	20	.4307	-1.3569	0.6017

Moderator Type	Term	Estimate	SE	t	df	p	95% CI (LB)	95% CI (UB)
	Unspecified Population	-0.4612	0.6955	-0.66	20	.5149	-1.9121	0.9897

Age, study quality, and study design significantly moderated the association between maladaptive personality traits and goal striving flexibility, with older participants and higher quality studies producing larger effect sizes and observational studies producing smaller effect sizes. In the combined model including all significant moderators, no predictors remained significant.

SI1.11 maladaptive personality traits

Moderator Type	Term	Estimate	SE	t	df	p	95% CI (LB)	95% CI (UB)
**Age**	Intercept	-0.2448	0.0671	-3.65	5	.0148	-0.4173	-0.0724
	zAge	0.0049	0.0016	3.16	5	.0252	0.0009	0.0089
**Study quality**	Intercept	-0.2178	0.1077	-2.02	5	.0990	-0.4945	0.0589
	Higher quality	0.6132	0.1260	4.86	5	.0046	0.2892	0.9372
**Study Design**	Intercept	-0.1354	0.0687	-1.97	5	.1056	-0.3119	0.0410
	Observational	-0.1715	0.0475	-3.61	5	.0153	-0.2935	-0.0495
**Country**	Intercept	-0.1771	0.0847	-2.09	2	.1716	-0.5415	0.1872
	Canada	0.0302	0.0706	0.43	2	.7104	-0.2736	0.3341
	France	-0.1857	0.0916	-2.03	2	.1798	-0.5797	0.2083
	Iran	-0.4156	0.0987	-4.21	2	.0520	-0.8403	0.0090
	Unspecified	-0.1138	0.0964	-1.18	2	.3595	-0.5287	0.3012
**Population**	Intercept	-0.1469	0.0629	-2.33	4	.0799	-0.3217	0.0278
	Medical Condition	-0.1813	0.0575	-3.16	4	.0343	-0.3409	-0.0218
	Unspecified	-0.1440	0.0721	-2.00	4	.1165	-0.3441	0.0562

Moderator Type	Term	Estimate	SE	t	df	p	95% CI (LB)	95% CI (UB)
**All Moderators**	Intercept	0.2187	0.3043	0.72	3	.5242	-0.7497	1.1871
	zAge	-0.0242	0.0149	-1.63	3	.2012	-0.0715	0.0230
	Study quality	0.4821	0.3662	1.32	3	.2796	-0.6835	1.6476
	Observational Design	-0.7382	0.4177	-1.77	3	.1753	-2.0676	0.5911

No significant moderators of the agency inhibiting variables category emerged.

SI1.12 agency inhibiting variables

Moderator Type	Term	Estimate	SE	t	df	p	95% CI (LB)	95% CI (UB)
**Age**	—	—	—	—	—	—	—	—
**Study Quality**	Intercept	-0.1507	0.0569	-2.65	2	.1180	-0.3956	0.0943
	Higher quality	-0.0444	0.2140	-0.21	2	.8549	-0.9652	0.8764
**Study Design**	Intercept	-0.1573	0.0666	-2.36	2	.1419	-0.4438	0.1291
	Observational	0.0266	0.1281	0.21	2	.8549	-0.5245	0.5777
**Country**	Intercept	-0.1307	0.1094	-1.19	2	.3546	-0.6015	0.3400
	USA	-0.0266	0.1281	-0.21	2	.8549	-0.5777	0.5245
**Population**	Intercept	-0.1573	0.0666	-2.36	2	.1419	-0.4438	0.1291
	Unspecified	0.0266	0.1281	0.21	2	.8549	-0.5245	0.5777

Goal Disengagement Outcome Variables

Moderator analyses of the goal progression category revealed several significant factors influencing the effect sizes. Age was a significant moderator, with effects becoming less negative as participant age increased. Study design also significantly influenced results; lab-based studies showed significantly more negative effects compared to other designs. Country of the sample was a significant moderator overall, with samples from Canada, Germany, Netherlands, Philippines, Spain, Switzerland, UK, USA, and unspecified countries

showing stronger effects. Additionally, population type significantly moderated effects, with general population, medical condition, migrants, online samples, and students exhibiting more negative effect sizes. When we included all significant moderators in a combined model, the effects of individual moderators were largely attenuated, although the unspecified country category remained a significant predictor.

SI1.13 goal progression

Moderator Model	Term	Estimate	SE	t	df	95% CI Lower	95% CI Upper	Sig.
**Age**	Intercept	-0.1176	0.0341	-3.45	39	-0.1865	-0.0488	**
	Age (z)	0.0040	0.0011	3.77	39	0.0019	0.0062	***
**Study Quality**	Intercept	-0.1206	0.0332	-3.63	39	-0.1878	-0.0534	***
	Study quality	-0.0388	0.1308	-0.30	39	-0.3034	0.2257	
**Study Design**	Intercept	-0.0227	0.0903	-0.25	37	-0.2057	0.1604	
	Lab-Based	-0.2760	0.0947	-2.91	37	-0.4678	-0.0841	**
	Longitudinal	-0.0124	0.0894	-0.14	37	-0.1936	0.1688	
	Observational	-0.1555	0.0904	-1.72	37	-0.3387	0.0276	.
**Country**	Intercept	-0.3657	0.0600	-6.09	29	-0.4884	-0.2429	***
	Canada	0.5374	0.0572	9.40	29	0.4205	0.6543	***
	China	-0.0240	0.0791	-0.30	29	-0.1857	0.1378	
	Germany	0.2723	0.0697	3.91	29	0.1298	0.4149	***
	Multiple	0.0769	0.0645	1.19	29	-0.0551	0.2089	
	Netherlands	0.3262	0.1354	2.41	29	0.0494	0.6030	*
	Philippines	0.2562	0.0798	3.21	29	0.0929	0.4194	**
	Spain	0.2832	0.1022	2.77	29	0.0743	0.4922	**
	Switzerland	0.5021	0.0808	6.22	29	0.3369	0.6673	***
	UK	0.3574	0.0641	5.57	29	0.2263	0.4886	***
	Unspecified	0.4479	0.0555	8.07	29	0.3344	0.5615	***
	USA	0.2399	0.0510	4.71	29	0.1357	0.3441	***
**Population**	Intercept	0.1668	0.0586	2.85	32	0.0475	0.2861	**
	GenPop	-0.2779	0.0598	-4.65	32	-0.3998	-0.1561	***

Moderator Model	Term	Estimate	SE	t	df	95% CI Lower	95% CI Upper	Sig.
	Medical Condition	-0.2435	0.0810	-3.01	32	-0.4085	-0.0786	**
	Migrants	-0.3342	0.0838	-3.99	32	-0.5050	-0.1634	***
	Online	-0.4514	0.0611	-7.39	32	-0.5758	-0.3270	***
	Parents	-0.1834	0.0983	-1.87	32	-0.3836	0.0168	.
	Police Officers	-0.0710	0.0777	-0.91	32	-0.2293	0.0873	
	Student	-0.2921	0.0529	-5.52	32	-0.3999	-0.1843	***
	Young Adults/Adolescents	-0.1011	0.1200	-0.84	32	-0.3456	0.1435	
**All Significant Moderators**	Intercept	-0.2367	0.3241	-0.73	21	-0.9107	0.4373	
	Age (z)	0.0002	0.0133	0.01	21	-0.0275	0.0278	
	Study Design: Lab-Based	0.0605	0.4648	0.13	21	-0.9061	1.0271	
	Study Design: Longitudinal	0.4406	0.6622	0.67	21	-0.9365	1.8178	
	Study Design: Observational	0.3605	0.5688	0.63	21	-0.8225	1.5435	
	Country: Canada	-0.0627	0.7832	-0.08	21	-1.6914	1.5659	
	Country: China	0.0452	0.1723	0.26	21	-0.3132	0.4036	
	Country: Germany	0.1796	0.2479	0.72	21	-0.3360	0.6951	
	Country: Multiple	0.3485	0.3017	1.16	21	-0.2789	0.9760	
	Country: Netherlands	-0.2461	0.5239	-0.47	21	-1.3355	0.8434	
	Country: Philippines	-0.3373	0.4439	-0.76	21	-1.2604	0.5858	
	Country: Spain	-0.2092	0.5853	-0.36	21	-1.4264	1.0080	
	Country: Switzerland	0.4117	0.2058	2.00	21	-0.0162	0.8396	.
	Country: UK	0.3165	0.2636	1.20	21	-0.2316	0.8647	
	Country: Unspecified	0.5984	0.1265	4.73	21	0.3354	0.8615	***
	Country: USA	0.1984	0.1543	1.29	21	-0.1224	0.5193	
	Population: GenPop	-0.5313	0.3083	-1.72	21	-1.1725	0.1099	.

Moderator Model	Term	Estimate	SE	t	df	95% CI Lower	95% CI Upper	Sig.
	Population: Online	-0.5750	0.4645	-1.24	21	-1.5410	0.3909	
	Population: Police Officers	-0.0127	0.3599	-0.04	21	-0.7612	0.7358	
	Population: Student	-0.5439	0.3273	-1.66	21	-1.2246	0.1369	

Study quality, sample country, and sample population significantly moderated the relation between goal disengagement and illbeing. Higher study quality was associated with smaller effect sizes. There were larger effects for studies conducted in Germany, Spain, multiple countries, the UK, USA, and unspecified locations. Population type also significantly moderated effects, with business owners/managers, caregivers, medical conditions, police officers, and elderly samples showing smaller or negative effects, whereas online populations showed significantly larger effects. When we simultaneously included all significant moderators, most lost significance except for Spain and online populations.

SI1.14 illbeing

Moderator Model	Term	Estimate	SE	t	df	95% CI Lower	95% CI Upper	Sig.
**Age**	Intercept	-0.1566	0.0856	-1.83	121	-0.3261	0.0129	.
	Age (z)	0.0001	0.0004	0.28	121	-0.0006	0.0008	
**Study Quality**	Intercept	-0.1548	0.0846	-1.83	121	-0.3223	0.0127	.
	Study quality	-0.1240	0.0538	-2.30	121	-0.2305	-0.0174	*
**Study Design**	Intercept	-0.2464	0.1024	-2.41	119	-0.4491	-0.0436	*
	Lab-Based	0.0334	0.1411	0.24	119	-0.2460	0.3128	
	Longitudinal	0.0808	0.0703	1.15	119	-0.0584	0.2201	
	Observational	0.1114	0.0694	1.60	119	-0.0261	0.2488	
**Country**	Intercept	-0.3469	0.0859	-4.04	111	-0.5170	-0.1767	***

Moderator Model	Term	Estimate	SE	t	df	95% CI Lower	95% CI Upper	Sig.
	Canada	0.0496	0.0370	1.34	111	-0.0238	0.1229	
	Germany	0.3672	0.0361	10.17	111	0.2957	0.4388	***
	Israel	0.1461	0.0739	1.98	111	-0.0004	0.2925	.
	Japan	0.1718	0.0742	2.32	111	0.0248	0.3189	*
	Multiple	0.2619	0.0398	6.58	111	0.1830	0.3408	***
	Netherlands	0.0014	0.0363	0.04	111	-0.0704	0.0733	
	Russia	0.1446	0.0442	3.27	111	0.0570	0.2321	**
	Spain	0.7712	0.0562	13.72	111	0.6599	0.8826	***
	UK	0.2610	0.0404	6.46	111	0.1809	0.3410	***
	Unspecified	0.1949	0.0373	5.23	111	0.1210	0.2688	***
	USA	0.2023	0.0333	6.07	111	0.1363	0.2683	***
**Population**	Intercept	-0.1053	0.1068	-0.99	114	-0.3168	0.1062	
	Business Owners/Managers	-0.3387	0.0748	-4.53	114	-0.4869	-0.1905	***
	Caregivers	-0.1898	0.0729	-2.60	114	-0.3342	-0.0453	*
	Elderly	-0.0523	0.0657	-0.80	114	-0.1824	0.0778	
	GenPop	-0.0436	0.0631	-0.69	114	-0.1685	0.0814	
	Medical Condition	-0.1653	0.0634	-2.61	114	-0.2909	-0.0397	*
	Online	0.1215	0.0685	1.77	114	-0.0142	0.2572	.
	Police Officers	-0.1947	0.0843	-2.31	114	-0.3617	-0.0277	*
	Students	-0.0658	0.0629	-1.05	114	-0.1905	0.0589	
**All Significant Moderators**	Intercept	-0.3806	0.1989	-1.91	102	-0.7751	0.0138	.
	Study Quality	0.0853	0.3145	0.27	102	-0.5384	0.7090	
	Germany	0.2086	0.1462	1.43	102	-0.0814	0.4986	
	Spain	0.7590	0.1927	3.94	102	0.3768	1.1411	***

Moderator Model	Term	Estimate	SE	t	df	95% CI Lower	95% CI Upper	Sig.
	Online	0.5025	0.1840	2.73	102	0.1376	0.8674	**

Study design significantly moderated the relation between goal disengagement and impairment. Although the overall country moderator was not significant, effects from studies conducted in South Korea were descriptively larger compared to others.

SI1.15 impairment

Moderator Type	Category	Estimate	SE	t	df	p	95% CI Lower	95% CI Upper	Significance
**Age**	Linear effect	-0.0011	0.0016	-0.66	9	0.5240	-0.0047	0.0026	
	Intercept	0.2329	0.0769	3.03	9	0.0143	0.0590	0.4069	*
**Study Quality**	Linear effect	-0.7522	0.3744	-2.01	9	0.0754	-1.5992	0.0948	.
	Intercept	0.2099	0.0664	3.16	9	0.0115	0.0597	0.3601	*
**Study Design**	Observational	0.2027	0.0788	2.57	9	0.0301	0.0244	0.3810	*
	Intercept	0.0893	0.0849	1.05	9	0.3205	-0.1029	0.2815	
**Country**	South Korea	0.3460	0.1005	3.44	6	0.0137	0.1001	0.5918	*
	Spain	0.1898	0.1050	1.81	6	0.1206	-0.0670	0.4466	
	Switzerland	0.1623	0.1154	1.41	6	0.2092	-0.1201	0.4446	
	USA	0.1969	0.0874	2.25	6	0.0652	-0.0170	0.4108	.
	Intercept	0.0541	0.1071	0.51	6	0.6313	-0.2080	0.3163	
**Population**	Medical Condition	-0.0235	0.0703	-0.33	8	0.7465	-0.1857	0.1387	
	Student	0.1443	0.0676	2.13	8	0.0654	-0.0116	0.3003	.
	Intercept	0.1997	0.1046	1.91	8	0.0926	-0.0414	0.4409	.

Goal Reengagement Outcome Variables

For the association between goal reengagement and illbeing, country was a significant moderator, with multiple countries differing significantly from the reference category. Specifically, studies from Canada, Germany, Scotland, Spain, the Netherlands, and unspecified countries showed significantly lower effect sizes compared to others. Population

subgroup was also a significant moderator, with business owners/managers, general population, medical condition groups, online samples, and students showing significantly lower effect sizes. When we simultaneously modeled all significant moderators, several country and population subgroups remained significant, confirming that both participant characteristics and study location contribute to variability in effect sizes.

SI1.16 illbeing

Moderator Type	Category	Estimate	SE	t	df	p	95% CI Lower	95% CI Upper	Significance
**Age**	Intercept	-0.0924	0.0264	-3.50	102	0.0007	-0.1448	-0.0400	***
	Moderator (Age)	0.0006	0.0004	1.41	102	0.1604	-0.0002	0.0014	
**Study Quality**	Intercept	-0.0962	0.0256	-3.76	102	0.0003	-0.1469	-0.0454	***
	Moderator (Study Quality)	-0.0569	0.0512	-1.11	102	0.2698	-0.1585	0.0448	
**Study Design**	Intercept	-0.0502	0.0739	-0.68	100	0.4982	-0.1968	0.0963	
	Lab Based	0.2052	0.1252	1.64	100	0.1043	-0.0432	0.4536	
	Longitudinal	-0.0608	0.0713	-0.85	100	0.3955	-0.2023	0.0806	
	Observational	-0.0564	0.0695	-0.81	100	0.4191	-0.1944	0.0815	
**Country**	Intercept	-0.0118	0.0670	-0.18	92	0.8605	-0.1449	0.1213	
	Canada	-0.1200	0.0579	-2.07	92	0.0409	-0.2349	-0.0051	*
	Germany	-0.3539	0.0992	-3.57	92	0.0006	-0.5510	-0.1568	***

Moderator Type	Category	Estimate	SE	t	df	p	95% CI Lower	95% CI Upper	Significance
	Japan	0.0601	0.0573	1.05	92	0.2968	-0.0537	0.1738	
	Multiple	0.0712	0.0581	1.23	92	0.2232	-0.0441	0.1865	
	Netherlands	-0.2044	0.0568	-3.60	92	0.0005	-0.3172	-0.0916	***
	Russia	-0.0706	0.0620	-1.14	92	0.2574	-0.1938	0.0525	
	Scotland	-0.2326	0.0867	-2.68	92	0.0087	-0.4048	-0.0604	**
	Spain	-0.4993	0.0704	-7.10	92	<.0001	-0.6391	-0.3596	***
	UK	-0.0834	0.0609	-1.37	92	0.1742	-0.2045	0.0376	
	Unspecified	-0.2050	0.0573	-3.58	92	0.0006	-0.3189	-0.0911	***
	USA	-0.1093	0.0556	-1.97	92	0.0524	-0.2197	0.0011	.
**Population**	Intercept	0.1436	0.0957	1.50	96	0.1368	-0.0463	0.3335	
	Business Owners/Managers	-0.2725	0.1069	-2.55	96	0.0124	-0.4846	-0.0604	*
	Caregivers	-0.1017	0.1403	-0.72	96	0.4705	-0.3802	0.1768	
	Elderly	-0.1497	0.0997	-1.50	96	0.1367	-0.3477	0.0483	
	General Population	-0.2282	0.0983	-2.32	96	0.0223	-0.4232	-0.0331	*

Moderator Type	Category	Estimate	SE	t	df	p	95% CI Lower	95% CI Upper	Significance
	Medical Condition	-0.3468	0.0984	-3.53	96	0.0007	-0.5420	-0.1515	***
	Online	-0.2138	0.1001	-2.14	96	0.0352	-0.4125	-0.0152	*
	Student	-0.3012	0.0982	-3.07	96	0.0028	-0.4962	-0.1062	**
**All Significant Moderators Combined**	Intercept	0.3305	0.1965	1.68	85	0.0962	-0.0601	0.7211	.
	Canada	-0.1695	0.1389	-1.22	85	0.2255	-0.4456	0.1066	
	Germany	-0.3467	0.1927	-1.80	85	0.0755	-0.7298	0.0364	.
	Japan	0.0655	0.1881	0.35	85	0.7286	-0.3084	0.4394	
	Multiple	0.0712	0.1586	0.45	85	0.6546	-0.2442	0.3866	
	Netherlands	-0.3195	0.1447	-2.21	85	0.0299	-0.6071	-0.0319	*
	Russia	-0.0706	0.1625	-0.43	85	0.6648	-0.3937	0.2524	
	Scotland	-0.3120	0.1941	-1.61	85	0.1118	-0.6980	0.0741	
	Spain	-0.5836	0.1672	-3.49	85	0.0008	-0.9161	-0.2511	***
	UK	-0.1843	0.1440	-1.28	85	0.2042	-0.4706	0.1021	

Moderator Type	Category	Estimate	SE	t	df	p	95% CI Lower	95% CI Upper	Significance
	Unspecified	-0.2507	0.1485	-1.69	85	0.0951	-0.5461	0.0446	.
	USA	-0.1901	0.1318	-1.44	85	0.1528	-0.4522	0.0719	
	Business Owners/Managers	-0.3391	0.1911	-1.77	85	0.0796	-0.7190	0.0408	.
	Caregivers	-0.1804	0.2140	-0.84	85	0.4017	-0.6059	0.2451	
	Elderly	-0.2915	0.1609	-1.81	85	0.0737	-0.6115	0.0286	.
	General Population	-0.3856	0.1563	-2.47	85	0.0156	-0.6963	-0.0749	*
	Medical Condition	-0.2991	0.1467	-2.04	85	0.0445	-0.5908	-0.0075	*
	Online	-0.3910	0.1639	-2.39	85	0.0192	-0.7168	-0.0652	*
	Student	-0.3062	0.1538	-1.99	85	0.0496	-0.6119	-0.0005	*

Further, age, study quality, sample country, and study design significantly moderated the association between goal reengagement and wellbeing. Older participant samples evinced slightly larger effect sizes. Higher quality studies also demonstrated notably larger effects. Observational studies yielded significantly smaller effect sizes compared to other designs. Samples from Spain, Switzerland, and USA displayed larger effects, whereas samples from other countries did not differ significantly. When we simultaneously modeled all significant moderators, we observed no significant combined effects.

SI1.17 wellbeing

Moderator	Category	Estimate	SE	t-value	df	p-value	CI Lower	CI Upper	Significance
**Age Moderator**	Intercept	0.2409	0.0444	5.4303	63	<.0001	0.1522	0.3295	***
	Age (continuous)	0.0035	0.0009	4.1384	63	0.0001	0.0018	0.0052	***
**Study Quality Moderator**	Intercept	0.2444	0.0439	5.5744	63	<.0001	0.1568	0.3321	***
	Study Quality (continuous)	0.3381	0.0908	3.7249	63	0.0004	0.1567	0.5194	***
**Study Design Moderator**	Intercept	0.3212	0.0498	6.4453	63	<.0001	0.2216	0.4208	***
	Observational vs. Other	-0.0987	0.0296	-3.3344	63	0.0014	-0.1578	-0.0395	**
**Country Moderator**	Intercept	0.1091	0.0995	1.0964	55	0.2777	-0.0903	0.3085	
	Canada	0.1853	0.0933	1.9858	55	0.0520	-0.0017	0.3724	.
	Japan	0.0781	0.1031	0.7580	55	0.4517	-0.1284	0.2847	
	Multiple Countries	0.1589	0.0889	1.7868	55	0.0795	-0.0193	0.3370	.
	Netherlands	0.1400	0.0840	1.6668	55	0.1012	-0.0283	0.3083	
	Spain	0.4130	0.0990	4.1735	55	0.0001	0.2147	0.6114	***
	Switzerland	0.2496	0.0982	2.5412	55	0.0139	0.0528	0.4464	*
	UK	0.1934	0.1357	1.4248	55	0.1599	-0.0786	0.4654	
	Unspecified Country	-0.0336	0.1046	-0.3208	55	0.7496	-0.2433	0.1761	
	USA	0.1760	0.0821	2.1446	55	0.0364	0.0115	0.3405	*

Moderator	Category	Estimate	SE	t-value	df	p-value	CI Lower	CI Upper	Significance
**Population Moderator**	Intercept	0.3143	0.1038	3.0287	58	0.0037	0.1066	0.5221	**
	Elderly	0.1005	0.1455	0.6904	58	0.4927	-0.1909	0.3918	
	General Population	-0.0406	0.0919	-0.4416	58	0.6604	-0.2246	0.1434	
	Medical Condition	-0.0074	0.0935	-0.0792	58	0.9371	-0.1945	0.1797	
	Online	-0.1121	0.1018	-1.1016	58	0.2752	-0.3158	0.0916	
	Student	-0.0880	0.0962	-0.9146	58	0.3642	-0.2805	0.1046	
	Young Adult/Adolescent	-0.1258	0.1177	-1.0684	58	0.2898	-0.3615	0.1099	
**All Significant Moderators Model**	Intercept	0.1571	0.2682	0.5859	52	0.5605	-0.3810	0.6953	
	Age	-0.0013	0.0034	-0.3828	52	0.7035	-0.0081	0.0055	
	Study Quality	0.4016	0.5033	0.7979	52	0.4286	-0.6084	1.4116	
	Observational Design	-0.0054	0.1337	-0.0403	52	0.9680	-0.2736	0.2629	
	Canada	0.0351	0.2578	0.1362	52	0.8922	-0.4822	0.5524	
	Japan	0.0506	0.2363	0.2143	52	0.8311	-0.4235	0.5248	
	Multiple Countries	0.2459	0.2687	0.9150	52	0.3644	-0.2933	0.7851	

Moderator	Category	Estimate	SE	t-value	df	p-value	CI Lower	CI Upper	Significance
	Netherlands	0.0721	0.2510	0.2874	52	0.7750	-0.4315	0.5757	
	Spain	0.4905	0.2706	1.8126	52	0.0757	-0.0525	1.0336	.
	Switzerland	0.1295	0.2900	0.4465	52	0.6571	-0.4525	0.7115	
	UK	0.1278	0.3544	0.3607	52	0.7198	-0.5833	0.8390	
	Unspecified Country	-0.2113	0.2868	-0.7369	52	0.4645	-0.7868	0.3641	
	USA	0.0933	0.2524	0.3696	52	0.7132	-0.4133	0.5998	

Goal Striving Flexibility Outcome Variables

Study design was a significant moderator of the association between goal striving flexibility and goal attainment, with both longitudinal and observational studies showing significantly smaller effects than experimental designs. Country of data collection also significantly moderated results: studies conducted in China, Iran, the Philippines, Spain, and Switzerland yielded significantly lower effect sizes. Population type moderated the results as well, with student samples associated with significantly higher effect sizes than other populations. A model including all significant moderators confirmed the unique influence of study design, country, and population on effect sizes, especially highlighting Switzerland (lower effects) and student populations (higher effects).

SI1.18 goal attainment

Moderator Model	Term	Estimate	SE	t-value	df	CI Lower	CI Upper	Significance
**Age**	Intercept	0.2235	0.1174	1.9032	23	-0.0194	0.4663	.

Moderator Model	Term	Estimate	SE	t-value	df	CI Lower	CI Upper	Significance
	Age	0.0007	0.0012	0.5878	23	-0.0017	0.0031	
**Study Quality**	Intercept	0.2211	0.1155	1.9143	23	-0.0178	0.4601	.
	Study Quality	-0.0808	0.1647	-0.4902	23	-0.4216	0.2600	
**Study Design**	Intercept	0.5039	0.1433	3.5167	22	0.2067	0.8010	**
	Longitudinal	-0.2330	0.0946	-2.4617	22	-0.4292	-0.0367	*
	Observational	-0.3213	0.0925	-3.4727	22	-0.5131	-0.1294	**
**Country**	Intercept	0.3967	0.1361	2.9150	16	0.1082	0.6852	*
	China	-0.1355	0.0617	-2.1956	16	-0.2664	-0.0047	*
	Germany	-0.1175	0.0790	-1.4866	16	-0.2850	0.0500	
	Iran	-0.2504	0.0686	-3.6520	16	-0.3957	-0.1050	**
	Philippines	-0.2731	0.1066	-2.5612	16	-0.4992	-0.0471	*
	Spain	-0.2024	0.0741	-2.7303	16	-0.3595	-0.0452	*
	Switzerland	-0.6184	0.1542	-4.0105	16	-0.9453	-0.2915	**
	Unspecified	-0.1532	0.0654	-2.3432	16	-0.2918	-0.0146	*
	USA	-0.0868	0.0653	-1.3308	16	-0.2252	0.0515	
**Population**	Intercept	0.1459	0.0767	1.9026	19	-0.0146	0.3063	.
	General Population	0.0073	0.0665	0.1102	19	-0.1319	0.1466	
	Medical Condition	-0.0521	0.0675	-0.7719	19	-0.1935	0.0892	
	Online	0.0037	0.0892	0.0410	19	-0.1831	0.1905	
	Student	0.3372	0.0821	4.1060	19	0.1653	0.5090	***
	Young Adult / Adolescent	-0.1121	0.0743	-1.5079	19	-0.2676	0.0435	

Moderator Model	Term	Estimate	SE	t-value	df	CI Lower	CI Upper	Significance
**All Significant Moderators Combined**	Intercept	0.5034	0.2510	2.0057	11	-0.0490	1.0559	.
	Study Design: Longitudinal	-0.4191	0.2105	-1.9910	11	-0.8825	0.0442	.
	Study Design: Observational	-0.2326	0.1850	-1.2575	11	-0.6398	0.1746	
	Country: China	-0.1135	0.1173	-0.9681	11	-0.3716	0.1446	
	Country: Germany	0.0059	0.1877	0.0316	11	-0.4071	0.4190	
	Country: Iran	-0.4977	0.2323	-2.1424	11	-1.0091	0.0136	.
	Country: Philippines	-0.1052	0.1842	-0.5710	11	-0.5107	0.3003	
	Country: Spain	-0.4622	0.2278	-2.0293	11	-0.9635	0.0391	.
	Country: Switzerland	-0.8035	0.2270	-3.5401	11	-1.3031	-0.3039	**
	Country: Unspecified	-0.0943	0.1423	-0.6628	11	-0.4075	0.2189	
	Country: USA	-0.3026	0.1796	-1.6849	11	-0.6980	0.0927	
	Population: General Population	0.1221	0.1004	1.2157	11	-0.0989	0.3431	
	Population: Medical Condition	0.2217	0.1908	1.1618	11	-0.1983	0.6416	
	Population: Student	0.6976	0.2027	3.4416	11	0.2515	1.1438	**

Significant moderators of the association between goal striving flexibility and functioning included study quality, study design, country, and population type. Studies rated higher in study quality reported substantially smaller effect sizes compared to lower-quality studies. Study design also moderated the findings: studies using an observational design yielded significantly larger effects than experimental or RCT-based studies. Country of data collection significantly influenced outcomes, with studies from Germany, the Netherlands, and USA reporting smaller effects, whereas studies from the Philippines and Spain showed

larger effects. Population type further moderated effects, with studies conducted in the general population and student samples showing reduced effect sizes. Notably, the full model including all significant moderators did not yield an overall significant moderator effect.

SI1.19 functioning

Moderator Model	Term	Estimate	SE	t-value	df	CI Lower	CI Upper	Significance
**Age**	Intercept	0.2344	0.1584	1.4803	41	-0.0854	0.5542	
	Age	-0.0004	0.0002	-1.5930	41	-0.0008	0.0001	
**Study Quality**	Intercept	0.2550	0.0856	2.9783	41	0.0821	0.4279	**
	Study Quality	-1.6426	0.0852	-19.2794	41	-1.8147	-1.4705	***
**Study Design**	Intercept	0.1978	0.1499	1.3202	40	-0.1050	0.5007	
	Observational	0.0528	0.0110	4.8070	40	0.0306	0.0750	***
	RCT	-0.0416	0.0938	-0.4436	40	-0.2313	0.1480	
**Country**	Intercept	0.2592	0.0521	4.9724	35	0.1534	0.3650	***
	France	0.0293	0.0552	0.5307	35	-0.0828	0.1414	
	Germany	-0.2421	0.0215	-11.2368	35	-0.2859	-0.1984	***
	Ireland	-0.1096	0.0873	-1.2561	35	-0.2868	0.0676	
	Netherlands	-0.6827	0.0822	-8.3102	35	-0.8495	-0.5159	***
	Philippines	0.1506	0.0285	5.2801	35	0.0927	0.2085	***
	Spain	0.1378	0.0665	2.0729	35	0.0028	0.2727	*
	USA	-0.2441	0.0616	-3.9603	35	-0.3692	-0.1190	***
**Population**	Intercept	0.3797	0.1800	2.1089	39	0.0155	0.7438	*
	General Population	-0.1917	0.0219	-8.7562	39	-0.2359	-0.1474	***
	Medical Condition	-0.0528	0.0332	-1.5916	39	-0.1200	0.0143	
	Student	-0.1459	0.0219	-6.6777	39	-0.1901	-0.1017	***
**All Significant Moderators Model**	Intercept	0.1548	0.2293	0.6752	30	-0.3134	0.6230	

Moderator Model	Term	Estimate	SE	t-value	df	CI Lower	CI Upper	Significance
	Study Quality	1.7605	2.3270	0.7566	30	-2.9919	6.5130	
	Study Design: Observational	0.2783	0.2625	1.0601	30	-0.2578	0.8144	
	Study Design: RCT	0.7893	0.5068	1.5574	30	-0.2458	1.8244	
	Country: France	-0.6473	0.4860	-1.3319	30	-1.6397	0.3452	
	Country: Germany	-0.6589	0.4377	-1.5056	30	-1.5528	0.2349	
	Country: Ireland	-0.7054	0.7166	-0.9843	30	-2.1690	0.7582	
	Country: Netherlands	-1.6650	0.8347	-1.9948	30	-3.3697	0.0396	.
	Country: Philippines	-0.0229	0.2641	-0.0867	30	-0.5623	0.5165	
	Country: Spain	-0.8537	0.7487	-1.1403	30	-2.3829	0.6754	
	Country: USA	-1.2094	0.6245	-1.9366	30	-2.4847	0.0660	.
	Population: General Population	0.1612	0.2346	0.6869	30	-0.3180	0.6404	
	Population: Medical Condition	0.6410	0.5358	1.1963	30	-0.4533	1.7354	

In addition, significant moderators of the association between goal flexibility and wellbeing included age, study quality, study design, country, and population. Specifically, studies involving older participants reported slightly larger effects, suggesting a small age-related increase in effect size. Higher study quality was associated with smaller effects, indicating that more rigorously conducted studies yielded more conservative estimates. Observational study designs showed significantly larger effects compared to other designs. Country of data collection was also a strong moderator: effect sizes were significantly lower in studies conducted in Canada, Finland, France, Germany, Ireland, Switzerland, thecNetherlands, USA, and those where country was unspecified. Conversely, effect sizes were significantly higher in Iran. For population type, studies involving student samples and

young adults/adolescents yielded smaller effects than the reference group, whereas general population, online samples, and medical condition groups did not differ significantly. In the model including all significant moderators, only the effect for Switzerland remained significant.

SI1.20 wellbeing

Moderator Model	Term	Estimate	SE	t-value	df	CI Lower	CI Upper	Significance
**Age**	Intercept	0.4185	0.0372	11.2572	97	0.3447	0.4923	***
	Age	0.0005	0.0002	2.1341	97	0.0000	0.0009	*
**Study Quality**	Intercept	0.4128	0.0356	11.6118	97	0.3423	0.4834	***
	Study Quality	-0.1445	0.0389	-3.7103	97	-0.2218	-0.0672	***
**Study Design**	Intercept	0.3639	0.0361	10.0936	97	0.2923	0.4355	***
	Observational	0.0647	0.0078	8.2576	97	0.0491	0.0802	***
**Country**	Intercept	0.6455	0.0480	13.4575	84	0.5501	0.7409	***
	Belgium	-0.1234	0.0713	-1.7318	84	-0.2651	0.0183	.
	Canada	-0.4553	0.1254	-3.6296	84	-0.7047	-0.2058	***
	China	-0.0146	0.0487	-0.3007	84	-0.1115	0.0822	
	Finland	-0.8919	0.0839	-10.6331	84	-1.0587	-0.7251	***
	France	-0.3241	0.0470	-6.8997	84	-0.4175	-0.2307	***
	Germany	-0.3209	0.0397	-8.0759	84	-0.3999	-0.2418	***
	Iran	0.2330	0.0900	2.5873	84	0.0539	0.4120	*
	Ireland	-0.1944	0.0644	-3.0170	84	-0.3225	-0.0663	**
	Multiple	-0.0571	0.0460	-1.2414	84	-0.1486	0.0344	
	Netherlands	-0.1674	0.0471	-3.5551	84	-0.2611	-0.0738	***
	Spain	0.0610	0.0581	1.0506	84	-0.0545	0.1765	
	Switzerland	-0.9760	0.1456	-6.7046	84	-1.2654	-0.6865	***

Moderator Model	Term	Estimate	SE	t-value	df	CI Lower	CI Upper	Significance
	Unspecified	-0.3021	0.0440	-6.8719	84	-0.3895	-0.2147	***
	USA	-0.4044	0.0421	-9.6164	84	-0.4880	-0.3208	***
**Population**	Intercept	0.4411	0.0408	10.7986	93	0.3600	0.5222	***
	General Population	-0.0332	0.0176	-1.8805	93	-0.0682	0.0019	.
	Medical Condition	0.0131	0.0218	0.6010	93	-0.0303	0.0565	
	Online	0.0476	0.0294	1.6178	93	-0.0108	0.1060	
	Student	-0.1812	0.0191	-9.5100	93	-0.2190	-0.1434	***
	Young Adult/Adolescent	-0.2408	0.0509	-4.7269	93	-0.3419	-0.1396	***
**All Significant Moderators**	Intercept	0.3173	0.2372	1.3376	76	-0.1551	0.7897	
	Age	0.0023	0.0042	0.5515	76	-0.0060	0.0106	
	Study Quality	0.9225	0.6466	1.4268	76	-0.3652	2.2103	
	Study Design: Observational	0.0385	0.1006	0.3832	76	-0.1617	0.2388	
	Country: Belgium	-0.1698	0.2482	-0.6841	76	-0.6640	0.3245	
	Country: Canada	-0.3325	0.3332	-0.9978	76	-0.9961	0.3312	
	Country: China	0.1712	0.2530	0.6765	76	-0.3328	0.6751	
	Country: Finland	-0.6671	0.3798	-1.7563	76	-1.4236	0.0894	.
	Country: France	-0.1598	0.2518	-0.6349	76	-0.6613	0.3416	
	Country: Germany	-0.1781	0.1913	-0.9306	76	-0.5591	0.2030	
	Country: Iran	0.6699	0.4178	1.6033	76	-0.1623	1.5021	
	Country: Ireland	-0.1793	0.2213	-0.8102	76	-0.6202	0.2615	
	Country: Multiple	0.3323	0.2838	1.1709	76	-0.2329	0.8976	

Moderator Model	Term	Estimate	SE	t-value	df	CI Lower	CI Upper	Significance
	Country: Netherlands	-0.2369	0.1964	-1.2066	76	-0.6281	0.1542	
	Country: Spain	0.0603	0.2442	0.2471	76	-0.4261	0.5468	
	Country: Switzerland	-0.9340	0.3483	-2.6812	76	-1.6277	-0.2402	**
	Country: Unspecified	0.0274	0.2841	0.0963	76	-0.5385	0.5933	
	Country: USA	-0.2578	0.2184	-1.1805	76	-0.6928	0.1772	
	Population: General Population	0.1188	0.1843	0.6446	76	-0.2483	0.4858	
	Population: Medical Condition	0.1295	0.1622	0.7982	76	-0.1936	0.4525	
	Population: Online	0.0316	0.2417	0.1306	76	-0.4499	0.5130	
	Population: Student	-0.0775	0.2819	-0.2747	76	-0.6390	0.4841	
	Population: Young Adult/Adolescent	-0.2341	0.3742	-0.6255	76	-0.9794	0.5112	

Search Terms (SI-2)

We will use the following search terms (as free text and keywords):

Include the key term “goal” with a 4 word adjacency search with the terms: (“disengag*” OR “reengag*” OR “unattain*” OR “dis-engag*” OR “re-engag*” OR "give up" OR “adjust*” OR “flexib*”)

<u>Web of Science</u>

TS=(“goal” NEAR/4 (“disengag*” OR “reengag*” OR “unattain*” OR “dis-engag*” OR “re-engag*” OR "give up" OR “adjust*” OR “flexib*”))

<u>Scopus</u>

Note: use advanced search

TITLE-ABS-KEY (“goal” W/4 (“disengag*” OR “reengag*” OR “unattain*” OR “dis-engag*” OR “re-engag*” OR "give up" OR “adjust*” OR “flexib*”))

<u>PsycInfo</u>

Note: use advanced search; Limit to English language

(goal adj4 (“disengag*” OR “reengag*” OR “unattain*” OR “dis-engag*” OR “re-engag*” OR "give up" OR “adjust*” OR “flexib*”)).mp [mp=title, abstract, heading word, table of contents, key concepts, original title, tests & measures, mesh]

<u>Business Source Ultimate</u>

Note: use advanced search; Limit language to English; limit to academic journals

(goal N4 (“disengag*” OR “reengag*” OR “unattain*” OR “dis-engag*” OR “re-engag*” OR "give up" OR “adjust*” OR “flexib*”) [TX All Text]

Exclude subject areas: Policy; climate change

<u>Proquest Dissertations and Theses Global</u>

Note: limit language to English; exclude by subject (computer science, electrical engineering, mechanical engineering, industrial engineering, British and irish literature, computer engineering, cellular biology, law, american literature, artificial intelligence, ecology, American history, chemical engineering, electromagnetics, engineering, film studies, finance, fine arts, information technology, international law)

NOFT(goal NEAR/4 (“disengag*” OR “reengag*” OR “unattain*” OR “dis-engag*” OR “re-engag*” OR "give up" OR “adjust*” OR “flexib*”)

<u>Medline</u>

Note: limit language to English, limit subjects to humans

(“goal” adj4 (“disengag*” OR “reengag*” OR “unattain*” OR “dis-engag*” OR “re-engag*” OR "give up" OR “adjust*” OR “flexib*”)).mp

Studies Included in Meta-Analysis (SI-3)

Abraham, J. (1993) *Successful aging in the workplace: An investigation of two approaches to developmental adaptation*. Doctoral Thesis, University of Tulsa. ProQuest Dissertations and Theses Global. https://www.proquest.com/dissertations-theses/successful-aging-workplace-investigation-two/docview/304091706/se-2?accountid=10382

Alberts, J. M. (2002) *The effects of illness-related perceived threat to life goals on illness self-management efforts: The mediating role of goal-related emotions and plans* [Doctoral dissertation, University of California Irvine]. ProQuest Dissertations & Theses. https://www.proquest.com/pqdtglobal1/dissertations-theses/effects-illness-related-perceived-threat-life/docview/304798572/sem-2

Albrecht, C.-M., Hattula, S., & Lehmann, D. R. (2017). The relationship between consumer shopping stress and purchase abandonment in task-oriented and recreation-oriented consumers. *Journal of the Academy of Marketing Science*, *45*(5), 720–740. https://doi.org/10.1007/s11747-016-0514-5

Allen, H. L., Gmelin, T., Moored, K. D., Boudreau, R. M., Smagula, S. F., Cohen, R. W., Katz, R., Stone, K., Cauley, J. A., Glynn, N. W., & and for the Osteoporotic Fractures in Men Study (MrOS) Research Group. (2022). Relationship between personality measures and perceived mental fatigability. *Journal of Aging and Health*, *34*(4–5), 750–760. https://doi.org/10.1177/08982643211055032

Arends, R. Y., Bode, C., Taal, E., & Van de Laar, M. A. F. J. (2013). The role of goal management for successful adaptation to arthritis. *Patient Education and Counseling*, *93*(1), 130–138. https://doi.org/10.1016/j.pec.2013.04.022

Asano, K., Ishimura, I., & Kodama, M. (2014). The functional role of resignation orientation on goal engagement, self-esteem, life satisfaction, and depression. *Health Psychology Research*, *2*(3), Article 1882. https://doi.org/10.4081/hpr.2014.1882

Bahrami, B., Mashhadi, A., Kareshki, H., & Bahrami, A. (2017). Role of mediating cognitive emotion regulation strategies and goal adjustment in relationship between personality

characteristics and quality of life of patients with cancer. *International Journal of Cancer Management*, *10*(12), Article e9317. https://doi.org/10.5812/ijcm.9317

Bailly, N., Hervé, C., Joulain, M., & Alaphilippe, D. (2012a). Validation of the French version of Brandtstädter and Renner's Tenacious Goal Pursuit (TGP) and Flexible Goal Adjustment (FGA) scales. *European Review of Applied Psychology*, *62*(1), 29–35. https://doi.org/10.1016/j.erap.2011.09.005

Bailly, N., Joulain, M., Hervé, C., & Alaphilippe, D. (2012b). Coping with negative life events in old age: The role of tenacious goal pursuit and flexible goal adjustment. *Aging & Mental Health*, *16*(4), 431–437. https://doi.org/10.1080/13607863.2011.630374

Barlow, M. A., Wrosch, C., Hamm, J., Sacher, T., Miller, G. E., & Kunzmann, U. (2022). Discrete negative emotions and goal disengagement in older adulthood: Context effects and associations with emotional well-being. *Emotion*, *22*(7), 1583–1594. https://doi.org/10.1037/emo0001091

Barlow, M. A., Wrosch, C., & Hoppmann, C. A. (2024). The interpersonal benefits of goal adjustment capacities: the sample case of coping with poor sleep in couples. *Frontiers in Psychology*, *15*, 1287470. https://doi.org/10.3389/fpsyg.2024.1287470

Bauer, I. (2004). *Unattainable goals across adulthood and old age: Benefits of goal adjustment capacities on well-being.* [Master's Thesis, Concordia University]. Spectrum Research Repository. https://spectrum.library.concordia.ca/id/eprint/8042/

Bernardo, A. B. I., Clemente, J. A. R., & Wang, T. Y. (2018). Working for a better future: Social mobility beliefs and expectations of Filipino migrant workers in Macau. *Australian Journal of Psychology, 70*(4), 350–360. https://doi.org/10.1111/ajpy.12209

Bernardo, A. B. I., Daganzo, M. A., & Burgos, J. F. C. (2022). Heritage culture detachment predicts hope and well-being of Filipino migrant workers: A study of low- and semi-skilled Filipino workers in Macau. *Psychological Studies, 67*(3), 372–384. https://doi.org/10.1007/s12646-022-00661-y

Bieleke, M., Wolff, W., & Keller, L. (2022). Getting trapped in a dead end? Trait self-control and boredom are linked to goal adjustment. *Motivation and Emotion, 46*, 837–851. https://doi.org/10.1007/s11031-022-09943-4.

Bock, J.-O., Hajek, A., & König, H.-H. (2018). The longitudinal association between psychological factors and health care use. *Health Services Research*, *53*(2), 1065–1091. https://doi.org/10.1111/1475-6773.12679

Boerner, K. (2004). Adaptation to disability among middle-aged and older adults: The role of assimilative and accommodative coping. *The Journals of Gerontology Series B: Psychological Sciences and Social Sciences*, *59*(1), P35–P42. https://doi.org/10.1093/geronb/59.1.P35

Bogdanoff, H. (2005) *The role of personal goals in predicting adjustment* and *well-being among individuals with acquired hearing loss* [Doctoral dissertation, American University]. ProQuest Dissertations & Theses. https://www.proquest.com/pqdtglobal1/dissertations-theses/role-personal-goals-predicting-adjustment-well/docview/305024753/sem-2

Borkoles, E., Kaiseler, M., Evans, A., Ski, C. F., Thompson, D. R., & Polman, R. C. J. (2018). Type D personality, stress, coping and performance on a novel sport task. *PLoS One*, *13*(4), Article e0196692. https://doi.org/10.1371/journal.pone.0196692

Boudrenghien, G., Frenay, M., & Bourgeois, É. (2012). Unattainable educational goals: Disengagement, reengagement with alternative goals, and consequences for subjective well-being. *European Review of Applied Psychology*, *62*(3), 147–159. https://doi.org/10.1016/j.erap.2012.04.002

Brandtstädter, J., & Renner, G. (1990). Tenacious goal pursuit and flexible goal adjustment: Explication and age-related analysis of assimilative and accommodative strategies of coping. *Psychology and Aging*, *5*(1), 58–67. https://doi.org/10.1037/0882-7974.5.1.58

Brun de Pontet, S. (2004). *Business and well being: The experience of entrepreneurs*. Master's Thesis, Concordia University. Spectrum Research Repository. https://spectrum.library.concordia.ca/id/eprint/8047/

Brun de Pontet, S. (2008). *Using theories of control and self-regulation to examine the leadership transition between a parent and child in family-owned business.* Doctoral Thesis, Concordia University. Spectrum Research Repository. https://spectrum.library.concordia.ca/id/eprint/975607/

Buchinger, L., Wahring, I. V., Ram, N., Hoppmann, C. A., Heckhausen, J., & Gerstorf, D. (2024). Kids or no kids? Life goals in one's 20s predict midlife trajectories of well-being.*Psychology and Aging, 39*(8), 897–914. https://doi.org/10.1037/pag0000862

Burr, A. (2006). *What's important: Personal values in the transition from work to retirement.* Doctoral Thesis, Concordia University. Spectrum Research Repository. https://spectrum.library.concordia.ca/id/eprint/9021/

Cappeliez, P., & Robitaille, A. (2010). Coping mediates the relationships between reminiscence and psychological well-being among older adults. *Aging & Mental Health*, *14*(7), 807–818. https://doi.org/10.1080/13607861003713307

Castonguay, A. L., Wrosch, C., & Sabiston, C. M. (2014). Systemic inflammation among breast cancer survivors: The roles of goal disengagement capacities and health-related self-protection: Inflammation in breast cancer survivors. *Psycho-Oncology*, *23*(8), 878–885. https://doi.org/10.1002/pon.3489

Catunda, C., Seidl, E. M. F., & Lemétayer, F. (2017). Illness perception and quality of life of HIV-positive persons: Mediation effects of tenacious and flexible goal pursuit. *Psychology, Health & Medicine*, *22*(2), 129–137. https://doi.org/10.1080/13548506.2016.1146404

Chan, C. C. (2022). Analyzing factors influencing college athletes' career actions using social cognitive career self-management model. *Journal of Career Development, 50*(5), 1058–1075. https://doi.org/10.1177/08948453221141441

Chang, E., & Lee, S. M. (2020). Mediating effect of goal adjustment on the relationship between socially prescribed perfectionism and academic burnout. *Psychology in the Schools*, *57*(2), 284–295. https://doi.org/10.1002/pits.22327

Chiesa, R., Antonio, A. A., Guglielmi, D., Mariani, M. G., & Mazzetti, G. (2024). Young adults' career goal management: The mediating role of perceived employability and career adaptability. *Australian Journal of Career Development*, *33*(1), 36-45. https://doi.org/10.1177/10384162231226079

Cho, Y. (2020). *Motivational regulation during post-stroke rehabilitation.* Master's Thesis, University of California Irvine. UC Irvine. https://escholarship.org/uc/item/0cq2t6g1

Cho, Y., Hamm, J., Heckhausen, J., & Cramer, S. (2024). Downward adjustment of rehabilitation goals may facilitate post-stroke arm motor recovery. *Psychology and Health, 40*(2), 175–191. https://doi.org/10.1080/08870446.2023.2211991

Cho, Y., Hamm, J. M., Heckhausen, J., & Cramer, S. C. (2022). The role of goal adjustment during rehabilitation from stroke. *Applied Psychology: Health and Well-Being*, *14*(1), 26–43. https://doi.org/10.1111/aphw.12288

Choi, H.-S., & Kim, S. Y. (2021). Crossing back over the Rubicon: Collectivistic value orientation and independent self-concept jointly promote effective goal revision in task groups. *Group Processes & Intergroup Relations*, *24*(6), 982–997. https://doi.org/10.1177/1368430220928123

Coffey, L. (2012) *Goals, adaptive self-regulation*, and *psychosocial adjustment to lower limb amputation*: *A longitudinal study* [Doctoral thesis, Dublin City University]. DCU Research Repository. http://doras.dcu.ie/17443/

Coffey, L., Gallagher, P., & Desmond, D. (2014a). Goal pursuit and goal adjustment as predictors of disability and quality of life among individuals with a lower limb amputation: A prospective study. *Archives of Physical Medicine and Rehabilitation*, *95*(2), 244–252. https://doi.org/10.1016/j.apmr.2013.08.011

Coffey, L., Gallagher, P., Desmond, D., & Ryall, N. (2014b). Goal pursuit, goal adjustment, and affective well-being following lower limb amputation. *British Journal of Health Psychology, 19*(2), 409–424. https://doi.org/10.1111/bjhp.12051

Čopková, R. (2022). Self-licensing and self-cleansing as self-regulation compensation mechanisms, and action crisis experiencing in the goal attainment. *Journal of Psychological and Educational Research*, *30*(1), 44–63.

Cornelius, A. (2024). *Motivation amidst uncertainty: The impact of self-regulatory processes on adaptation to change.* Doctoral Thesis, Saint Louis University. ProQuest Dissertations Publishing.

Corrigan, E. (2019). *Conditional goal setting and flexible goal adjustment in depression.* Doctoral Thesis, University of Exeter. Open Research Exeter. http://hdl.handle.net/10871/38948

Crane, C., Jandric, D., Barnhofer, T., & Williams, J. M. G. (2010). Dispositional mindfulness, meditation, and conditional goal setting. *Mindfulness*, *1*(4), 204–214. https://doi.org/10.1007/s12671-010-0029-y

Creed, P. A., & Blume, K. (2013). Compromise, well-being, and action behaviors in young adults in career transition. *Journal of Career Assessment*, *21*(1), 3–19. https://doi.org/10.1177/1069072712453830

Creed, P. A., & Hood, M. (2014). Disengaging from unattainable career goals and reengaging in more achievable ones. *Journal of Career Development*, *41*(1), 24–42. https://doi.org/10.1177/0894845312471195

Creed, P. A., Kjoelaas, S., & Hood, M. (2016). Testing a goal-orientation model of antecedents to career calling. *Journal of Career Development*, *43*(5), 398–412. https://doi.org/10.1177/0894845315603822

Darlington, A.-S. E., Dippel, D. W. J., Ribbers, G. M., Van Balen, R., Passchier, J., & Busschbach, J. J. V. (2007). Coping strategies as determinants of quality of life in stroke patients: A longitudinal study. *Cerebrovascular Diseases*, *23*(5–6), 401–407. https://doi.org/10.1159/000101463

Davis, M., Ventura, J. L., Wieners, M., Covington, S. N., Vanderhoof, V. H., Ryan, M. E., Koziol, D. E., Popat, V. B., & Nelson, L. M. (2010). The psychosocial transition associated with spontaneous 46,XX primary ovarian insufficiency: Illness uncertainty, stigma, goal flexibility, and purpose in life as factors in emotional health. *Fertility and Sterility*, *93*(7), 2321–2329. https://doi.org/10.1016/j.fertnstert.2008.12.122

Dempsey, R. C., Eardley, K., & Dodd, A. L. (2022). The role of tenacious versus flexible goal pursuit in the vulnerability to bipolar disorder. *Current Psychology*, *41*(4), 2382–2389. https://doi.org/10.1007/s12144-020-00748-7

Dhingra, K., Boduszek, D., & O’Connor, R. C. (2016). A structural test of the Integrated Motivational-Volitional model of suicidal behaviour. *Psychiatry Research*, *239*, 169–178. https://doi.org/10.1016/j.psychres.2016.03.023

Dickson, J., Hart, A., Fox-Harding, C., & Huntley, C. (2023). Adaptive goal processes and underlying motives that sustain mental wellbeing and new year exercise resolutions.

International Journal of Environmental Research and Public Health, *20*(2), Article 901. https://doi.org/10.3390/ijerph20020901

Dunne, E. (2006). *The short-term association between acute physical symptoms and negative affect in older adults: The buffering effects of goal adjustment capacities.* Master's Thesis, Concordia University. Spectrum Research Repository. https://spectrum.library.concordia.ca/id/eprint/9023/

Dutt, A. J., Gabrian, M., & Wahl, H.-W. (2016). Developmental Regulation and Awareness of Age-Related Change: A (Mostly) Unexplored Connection. *The Journals of Gerontology Series B: Psychological Sciences and Social Sciences, 73*(6), 944–953. https://doi.org/10.1093/geronb/gbw084

Dutt, A. J., Gabrian, M., & Wahl, H.-W. (2018). Awareness of age-related change and depressive symptoms in middle and late adulthood: Longitudinal associations and the role of self-regulation and calendar age. *The Journals of Gerontology Series B: Psychological Sciences and Social Sciences, 73*(6), 944–953. https://doi.org/10.1093/geronb/gbw095

Eddington, K. M. (2014). Perfectionism, goal adjustment, and self-regulation: A short-term follow-up study of distress and coping. *Self and Identity*, *13*(2), 197–213. https://doi.org/10.1080/15298868.2013.781740

Eddington, K. M., Burgin, C. J., & Majestic, C. (2016). Individual differences in expectancies for change in depression: Associations with goal pursuit and daily experiences. *Journal of Social and Clinical Psychology*, *35*(8), 629–642. https://doi.org/10.1521/jscp.2016.35.8.629

Eddington, K. M., Silvia, P. J., Foxworth, T. E., Hoet, A., & Kwapil, T. R. (2015). Motivational deficits differentially predict improvement in a randomized trial of self-system therapy for depression. *Journal of Consulting and Clinical Psychology*, *83*(3), 602–616. https://doi.org/10.1037/a0039058

Esteve, R., López-Martínez, A. E., Peters, M. L., Serrano-Ibáñez, E. R., Ruiz-Párraga, G. T., & Ramírez-Maestre, C. (2018). Optimism, positive and negative Affect, and goal Adjustment strategies: Their relationship to activity patterns in patients with chronic musculoskeletal pain. *Pain Research and Management*, *2018*, Article 6291719. https://doi.org/10.1155/2018/6291719

Fink, E. R. (2019). *The role of optimism in goal adjustment behavior and subjective well-being.* Doctoral Thesis, Indiana State University. Proquest Dissertations and Theses Global. https://www.proquest.com/dissertations-theses/role-optimism-goal-adjustment-behavior-subjective/docview/2239975038/se-2?accountid=10382

Fiske, A., Bamonti, P. M., Nadorff, M. R., Petts, R. A., & Sperry, J. A. (2013). Control strategies and suicidal ideation in older primary care patients with functional limitations. *The International Journal of Psychiatry in Medicine*, *46*(3), 271–289. https://doi.org/10.2190/PM.46.3.c

Frazier, L. D., Newman, F. L., & Jaccard, J. (2007). Psychosocial outcomes in later life: A multivariate model. *Psychology and Aging*, *22*(4), 676–689. https://doi.org/10.1037/0882-7974.22.4.676

Gagnè, M., Wrosch, C., & Brun De Pontet, S. (2011). Retiring from the family business: The role of goal adjustment capacities. *Family Business Review*, *24*(4), 292–304. https://doi.org/10.1177/0894486511410688

Garnefski, N., Grol, M., Kraaij, V., & Hamming, J. F. (2009a). Cognitive coping and goal adjustment in people with Peripheral Arterial Disease: Relationships with depressive symptoms. *Patient Education and Counseling*, *76*(1), 132–137. https://doi.org/10.1016/j.pec.2008.11.009

Garnefski, N., & Kraaji, V. (2010). Do cognitive coping and goal adjustment strategies used shortly after myocardial infarction predict depressive outcomes 1 year later? *Journal of Cardiovascular Nursing*, *25*(5), 383–389. https://doi.org/10.1097/JCN.0b013e3181d298ca

Garnefski, N., & Kraaij, V. (2012). Cognitive coping and goal adjustment are associated with symptoms of depression and anxiety in people with acquired hearing loss. *International Journal of Audiology*, *51*(7), 545–550. https://doi.org/10.3109/14992027.2012.675628

Garnefski, N., Kraaij, V., De Graaf, M., & Karels, L. (2010). Psychological intervention targets for people with visual impairments: The importance of cognitive coping and goal adjustment. *Disability and Rehabilitation*, *32*(2), 142–147. https://doi.org/10.3109/09638280903071859

Garnefski, N., Kraaij, V., Schroevers, M. J., Aarnink, J., van der Heijden, D. J., van Es, S. M., van Herpen, M., & Somsen, G. A. (2009b). Cognitive coping and goal adjustment after first-time

myocardial infarction: Relationships with symptoms of depression. *Behavioral Medicine, 35*(3), 79–86. https://doi.org/10.1080/08964280903232068

Garroway, A. M. (2015). *Resilience in Parkinson's Disease: An empirical examination of age-related components of the construct.* [Doctoral Thesis, Virginia Commonwealth University]. VSU Scholars Compass. https://doi.org/10.25772/07M8-2631

Goossens, M. E., Kindermans, H. P., Morley, S. J., Roelofs, J., Verbunt, J., & Vlaeyen, J. W. (2010). Self-discrepancies in work-related upper extremity pain: Relation to emotions and flexible-goal adjustment. *European Journal of Pain*, *14*(7), 764–770. https://doi.org/10.1016/j.ejpain.2009.11.012

Greve, W., Hauser, J., & Rühs, F. (2021a). Humorous coping with unrequited love: Is perspective change important? *Frontiers in Psychology, 12,* Article 653900. https://doi.org/10.3389/fpsyg.2021.653900

Greve, W., Koch, M., Rasche, V. & Kersten, K. (2021b). Extending the scope of the 'cognitive advantage' hypothesis: Multilingual individuals show higher flexibility of goal adjustment. *Journal of Multilingual and Multicultural Development, 45*(4), 822–838. https://doi.org/10.1080/01434632.2021.1922420

Greve, W., & Thomsen, T. (2016). Evolutionary advantages of free play during childhood. *Evolutionary Psychology*, *14*(4), Article 147470491667534. https://doi.org/10.1177/1474704916675349

Greve, W., Thomsen, T., & Dehio, C. (2014). Does playing pay? The fitness-effect of free play during childhood. *Evolutionary Psychology*, *12*(2), Article 147470491401200. https://doi.org/10.1177/147470491401200210

Grümer, S., Silbereisen, R. K., & Heckhausen, J. (2013). Subjective well-being in times of social change: Congruence of control strategies and perceived control. *International Journal of Psychology*, *48*(6), 1246–1259. https://doi.org/10.1080/00207594.2012.744839

Gživnová, V., & Kohútová, V. (2024). Proactive Coping as a Mediator in the Effect of Big Five Personality Traits on the Goal Restriction Reaction. *Studia Psychologica, 66*(2), 138-150. https://doi.org/10.31577/sp.2024.02.896

Haase, C. M., Aviram, T., Wrosch, C., Silbereisen, R. K., & Heckhausen, J. (2021). Well-being as a resource for goal reengagement: Evidence from two longitudinal studies. *Motivation Science*, *7*(2), 21–31. https://doi.org/10.1037/mot0000199

Haase, C. M., Heckhausen, J., & Silbereisen, R. K. (2012). The interplay of occupational motivation and well-being during the transition from university to work. *Developmental Psychology*, *48*(6), 1739–1751. https://doi.org/10.1037/a0026641

Haase, C. M., Heckhausen, J., & Wrosch, C. (2013). Developmental regulation across the life span: Toward a new synthesis. *Developmental Psychology*, *49*(5), 964–972. https://doi.org/10.1037/a0029231

Hajek, A., & König, H.-H. (2017). The role of flexible goal adjustment in the effect of informal caregiving on depressive symptoms: Evidence of a large population-based longitudinal study in Germany from 2002 to 2011. *Quality of Life Research*, *26*(2), 419–427. https://doi.org/10.1007/s11136-016-1399-y

Hajek, A., & König, H.-H. (2021). Flexible goal adjustment moderates the link between self-rated health and subjective well-being. Findings from the general population. *Aging & Mental Health*, *25*(7), 1345–1350. https://doi.org/10.1080/13607863.2020.1765313

Halkjelsvik, T. (2015). Substance use disinhibition associated with economically rational decisions to quit a boring task. *Personality and Individual Differences*, *80*, 101–106. https://doi.org/10.1016/j.paid.2015.02.028

Hamm, J., Barlow, M., Garcia, O., & Duggan, K. (2023). Context-dependent shifts in self-regulatory personality processes during COVID-19: Changes in control predict dynamic shifts in goal reengagement capacity. *Social and Personality Psychology Compass*, *17*(8), Article e12771. https://doi.org/10.1111/spc3.12771

Hamm, J. M., Heckhausen, J., Shane, J., & Lachman, M. E. (2020). Risk of cognitive declines with retirement: Who declines and why? *Psychology and Aging*, *35*(3), 449–457. https://doi.org/10.1037/pag0000453

Hamm, J. M., Tan, J. X., Barlow, M. A., Delaney, R. L., & Duggan, K. A. (2022). Goal adjustment capacities in uncontrollable life circumstances: Benefits for psychological well-

being during COVID-19. *Motivation and Emotion, 45*(3), 319–335. https://doi.org/10.1007/s11031-022-09941-6

Hanssen, M. M., Vancleef, L. M. G., Vlaeyen, J. W. S., Hayes, A. F., Schouten, E. G. W., & Peters, M. L. (2015). Optimism, motivational coping and well-being: Evidence supporting the importance of flexible goal adjustment. *Journal of Happiness Studies*, *16*(6), 1525–1537. https://doi.org/10.1007/s10902-014-9572-x

Haratsis, J. M., Creed, P. A., & Hood, M. (2015a). Measuring assimilative and accommodative resources in young adults: Development and initial validation of suitable scales. *Personality and Individual Differences*, *81*, 61–66. https://doi.org/10.1016/j.paid.2014.12.011

Haratsis, J. M., Creed, P. A., & Hood, M. (2016). Cross-lagged relationships between person-based resources, self-perceptions, and career and life satisfaction in young adults. *International Journal for Educational and Vocational Guidance*, *16*(2), 169–188. https://doi.org/10.1007/s10775-015-9301-y

Haratsis, J. M., Hood, M., & Creed, P. A. (2015b). Career goals in young adults: Personal resources, goal appraisals, attitudes, and goal management strategies. *Journal of Career Development*, *42*(5), 431–445. https://doi.org/10.1177/0894845315572019

Hartanto, A., Yee-Man Lau, I., & Yong, J. C. (2020). Culture moderates the link between perceived obligation and biological health risk: Evidence of culturally distinct pathways for positive health outcomes. *Social Science & Medicine*, *244*, Article 112644. https://doi.org/10.1016/j.socscimed.2019.112644

Hatzigeorgiadis, A. (2006). Approach and avoidance coping during task performance in young men: The role of goal attainment expectancies. *Journal of Sports Sciences*, *24*(3), 299–307. https://doi.org/10.1080/17461390500188645

Heinitz, K., Lorenz, T., Schulze, D., & Schorlemmer, J. (2018). Positive organizational behavior: Longitudinal effects on subjective well-being. *PLoS One*, *13*(6), Article e0198588. https://doi.org/10.1371/journal.pone.0198588

Henderson, M. D., Gollwitzer, P. M., & Oettingen, G. (2007). Implementation intentions and disengagement from a failing course of action. *Journal of Behavioral Decision Making*, *20*(1), 81–102. https://doi.org/10.1002/bdm.553

Henselmans, I., Fleer, J., van Sonderen, E., Smink, A., Sanderman, R., & Ranchor, A. V. (2011). The tenacious goal pursuit and flexible goal adjustment scales: A validation study. *Psychology and Aging, 26*(1), 174–180. https://doi.org/10.1037/a0021536

Herrmann, M., Baur, V., Brandstätter, V., Hänggi, J., & Jäncke, L. (2014). Being in two minds: The neural basis of experiencing action crises in personal long-term goals. *Social Neuroscience, 9*(6), 548–561. https://doi.org/10.1080/17470919.2014.933715

Herrmann, M., Brandstätter, V., & Wrosch, C. (2019). Downgrading goal-relevant resources in action crises: The moderating role of goal reengagement capacities and effects on well-being. *Motivation and Emotion*, *43*(4), 535–553. https://doi.org/10.1007/s11031-019-09755-z

Heyl, V., Wahl, H.-W., & Mollenkopf, H. (2007). Affective well-being in old age: The role of tenacious goal pursuit and flexible goal adjustment. *European Psychologist*, *12*(2), 119–129. https://doi.org/10.1027/1016-9040.12.2.119

Hoet, A. C. (2014). *The relationship between goal characteristics and emotional well-being*. Master's Thesis, University of North Carolina at Greensboro. Proquest Dissertations and Theses Global. https://www.proquest.com/dissertations-theses/relationship-between-goal-characteristics/docview/1616667780/se-2?accountid=10382

Holding, A. C., Hope, N. H., Harvey, B., Marion Jetten, A. S., & Koestner, R. (2017). Stuck in limbo: Motivational antecedents and consequences of experiencing action crises in personal goal pursuit. *Journal of Personality*, *85*(6), 893–905. https://doi.org/10.1111/jopy.12296

Holding, A., Moore, A., Verner-Filion, J., Kachanoff, F., & Koestner, R. (2022). Choosing to lose it: The role of autonomous motivation in goal disengagement. *Motivation and Emotion, 46*(6), 769–789. https://doi.org/10.1007/s11031-022-09952-3

Holton, E., Grohn, J., Ward, H., Manohar, S. G., O'reilly, J. X., & Kolling, N. (2024). Goal commitment is supported by vmPFC through selective attention. *Nature Human Behaviour, 8*(7), 1351-1365. https://doi.org/10.1038/s41562-024-01844-5

Hrabluik, C. (2009). *The dark side of goal setting: Examining the relationship between perfectionism and maximum versus typical employee performance*. Doctoral Thesis, University of Toronto. Library and Archives Canada

Hu, S., Creed, P. A., & Hood, M. (2017). Career goal revision in response to negative feedback: Testing a longitudinal cross-lagged model. *Journal of Counseling Psychology*, *64*(3), 335–345. https://doi.org/10.1037/cou0000193

Hu, S., Hood, M., & Creed, P. A. (2017). Negative career feedback and career goal disengagement in young adults: The moderating role of mind-set about work. *Journal of Vocational Behavior*, *102*, 63–71. https://doi.org/10.1016/j.jvb.2017.07.006

Hu, S., Creed, P. A., & Hood, M. (2019). Does socioeconomic status shape young people's goal revision processes in the face of negative career feedback? *Journal of Vocational Behavior*, *110*, 89–101. https://doi.org/10.1016/j.jvb.2018.11.011

Hubley, C., & Scholer, A. A. (2022). Melting COVID-frozen goals: How goal disengagement supports well-being during the COVID-19 pandemic. *Motivation and Emotion*, *46*(6), 752–768. https://doi.org/10.1007/s11031-022-09959-w

Huning, T. M. (2009). *Goal orientation and self-defeating Behavior: The mediative role of adaptive and maladaptive processes.* Doctoral Thesis, University of Memphis. Proquest Dissertations and Theses Global. https://www.proquest.com/dissertations-theses/goal-orientation-self-defeating-behavior/docview/304927106/se-2?accountid=10382

Ivanova, S., & Tornikoski, E. (2022). Termination of nascent entrepreneurship: The central effects of action crisis in new venture creation. *Journal of Small Business Management, 62*(2), 1–45. https://doi.org/10.1080/00472778.2022.2140160

Jawahar, I. M., & Shabeer, S. (2021). How does negative career feedback affect career goal disengagement? The mediating roles of career planning and psychological well-being. *Journal of Career Development*, *48*(4), 385–399. https://doi.org/10.1177/0894845319853637

Jobin, J., & Wrosch, C. (2016). Goal disengagement capacities and severity of disease across older adulthood: The sample case of the common cold. *International Journal of Behavioral Development*, *40*(2), 137–144. https://doi.org/10.1177/0165025415597549

Kappes, C., & Bermeitinger, C. (2016). The emotional Stroop as an emotion Regulation task. *Experimental Aging Research*, *42*(2), 161–194. https://doi.org/10.1080/0361073X.2016.1132890

Kappes, C., & Greve, W. (2024). Individual differences in goal adjustment: convergence and divergence among three theoretical models. *Frontiers in psychology*, *15*, 1288667. https://doi.org/10.3389/fpsyg.2024.1288667

*Kappes, C., Marion-Jetten, A. S., Taylor, G., Schad, D. J., Dreßler, B., El-Hayek, S., ... & Stach, P. (2023). The role of mindfulness and autonomous motivation for goal progress and goal adjustment: an intervention study. *Motivation and Emotion*, *47*(6), 946-964. https://doi.org/10.1007/s11031-023-10033-2

Kappes, C., & Thomsen, T. (2020). Imitation of goal engagement and disengagement processes in romantic relationships. *European Journal of Personality*, *34*(2), 234–244. https://doi.org/10.1002/per.2244

Khan, S. A., Tang, J., & Joshi, K. (2014). Disengagement of nascent entrepreneurs from the start-up process. *Journal of Small Business Management*, *52*(1), 39–58. https://doi.org/10.1111/jsbm.12032

Kleine, A., Schmitt, A., & Wisse, B. (2024). Challenge and threat appraisal of entrepreneurial errors: A latent profile analysis and examination of coping responses. *Current Psychology, 43*, 1206–1220. https://doi.org/10.1007/s12144-023-04370-1

Koch, M. J., & Greve, W. (2024). The benefits of cultural confrontation: Does the experience of cultural heterogeneity predict individual differences in accommodative regulation?. *International Journal of Intercultural Relations*, *102*, 102017. https://doi.org/10.1016/j.ijintrel.2024.102017

Koch, M., Greve, W., & Kersten, K. (2023). Extending the cognitive advantage hypothesis: A conceptual replication study of the relationship between multilingualism and flexible goal adjustment. *Journal of Multilingual and Multicultural Development, 46*(2), 455–469. https://doi.org/10.1080/01434632.2023.2189262

König, C. J., Van Eerde, W., & Burch, A. (2010). Predictors and consequences of daily goal adaptation: A diary study. *Journal of Personnel Psychology*, *9*(1), 50–56. https://doi.org/10.1027/1866-5888/a000002

Koppe, K., & Rothermund, K. (2017). Let it go: Depression facilitates disengagement from unattainable goals. *Journal of Behavior Therapy and Experimental Psychiatry*, *54*, 278–284. https://doi.org/10.1016/j.jbtep.2016.10.003

Kotter-Grühn, D., Scheibe, S., Blanchard-Fields, F., & Baltes, P. B. (2009). Developmental emergence and functionality of Sehnsucht (life longings): The sample case of involuntary childlessness in middle-aged women. *Psychology and Aging*, *24*(3), 634–644. https://doi.org/10.1037/a0016359

Kraaij, V., & Garnefski, N. (2012). Coping and depressive symptoms in adolescents with a chronic medical condition: A search for intervention targets. *Journal of Adolescence*, *35*(6), 1593–1600. https://doi.org/10.1016/j.adolescence.2012.06.007

Kraaij, V., & Garnefski, N. (2015). Cognitive, behavioral and goal adjustment coping and depressive symptoms in young people with diabetes: A search for intervention targets for coping skills training. *Journal of Clinical Psychology in Medical Settings*, *22*(1), 45–53. https://doi.org/10.1007/s10880-015-9417-8

Kraaij, V., Garnefski, N., & Schroevers, M. J. (2009). Coping, goal adjustment, and positive and negative affect in definitive infertility. *Journal of Health Psychology*, *14*(1), 18–26. https://doi.org/10.1177/1359105308097939

Kraaij, V., Garnefski, N., Schroevers, M. J., Van Der Veek, S. M. C., Witlox, R., & Maes, S. (2008a). Cognitive coping, goal self-efficacy and personal growth in HIV-infected men who have sex with men. *Patient Education and Counseling*, *72*(2), 301–304. https://doi.org/10.1016/j.pec.2008.04.007

Kraaij, V., Garnefski, N., Schroevers, M. J., Weijmer, J., & Helmerhorst, F. (2010). Cognitive coping, goal adjustment, and depressive and anxiety symptoms in people undergoing infertility treatment: A prospective study. *Journal of Health Psychology*, *15*(6), 876–886. https://doi.org/10.1177/1359105309357251

Kraaij, V., van der Veek, S. M. C., Garnefski, N., Schroevers, M., Witlox, R., & Maes, S. (2008b). Coping, goal adjustment, and psychological well-being in HIV-infected men who have sex with men. *AIDS Patient Care and STDs*, *22*(5), 395–402. https://doi.org/10.1089/apc.2007.0145

Kranz, D., Bollinger, A., & Nilges, P. (2010). Chronic pain acceptance and affective well-being: A coping perspective. *European Journal of Pain, 14*(10), 1021–1025. https://doi.org/10.1016/j.ejpain.2010.03.010

Kubicek, B., Korunka, C., Raymo, J. M., & Hoonakker, P. (2011). Psychological well-being in retirement: The effects of personal and gendered contextual resources. *Journal of Occupational Health Psychology, 16*(2), 230–246. https://doi.org/10.1037/a0022334

Lechner, C. M., Silbereisen, R. K., Tomasik, M. J., & Wasilewski, J. (2015). Getting going and letting go: Religiosity fosters opportunity-congruent coping with work-related uncertainties. *International Journal of Psychology, 50*(3), 205–214. https://doi.org/10.1002/ijop.12093

Leipold, B., & Loepthien, T. (2025). Accommodative coping and preparation for age-related changes: Results from a four-wave panel study. *International Journal of Behavioral Development,* 01650254241312144. https://doi.org/10.1177/01650254241312144

Leipold, B., Loepthien, T., Loidl, B., & Saalwirth, C. (2023). Goal adjustment and subjective well-being in adulthood: Longitudinal results from a three-wave panel study. *Journal of Individual Differences, 44*(4), 245–253. https://doi.org/10.1027/1614-0001/a000398

Liu, P., Thrasher, G., Ye, Y., & Shi, J. (2023). Goal-pursuit tendencies and retirement planning: A time-lagged and multi-dimensional investigation. *Work Aging and Retirement, 10*(3), 300–307. https://doi.org/10.1093/workar/waad002

Loidl, B., & Leipold, B. (2019). Facets of accommodative coping in adulthood. *Psychology and Aging, 34*(5), 640–654. https://doi.org/10.1037/pag0000378

Lyons, K. D., Wechsler, S. B., Ejem, D. B., Stevens, C. J., Azuero, A., Khalidi, S., ... & Bakitas, M. A. (2024). Telephone-based rehabilitation intervention to optimize activity participation after breast cancer: a randomized clinical trial. *JAMA Network Open, 7*(3), e242478. https://doi.org/10.1001/jamanetworkopen.2024.2478

Ma, R., Zhang, C., & Xu, W. (2025). The Moderating Role of Control Strategies on the Relationship between Negative Emotions and QoL in the Elderly: A Longitudinal Study. *International Journal of Mental Health Promotion, 27*(4). 469-483. https://doi.org/10.32604/ijmhp.2025.060351

Mahlo, L., & Windsor, T. D. (2021). Older and more mindful? Age differences in mindfulness components and well-being. *Aging & Mental Health, 25*(7), 1320–1331. https://doi.org/10.1080/13607863.2020.1734915

Majestic, C. (2016). *A motivational perspective on caregiver psychological distress*. Doctoral Thesis, University of North Carolina at Greensboro]. Proquest Dissertations and Theses. https://www.proquest.com/dissertations-theses/motivational-perspective-on-caregiver/docview/1817015047/se-2?accountid=10382

Majestic, C., & Eddington, K. M. (2019). The impact of goal adjustment and caregiver burden on psychological distress among caregivers of cancer patients. *Psycho-Oncology*, *28*(6), 1293–1300. https://doi.org/10.1002/pon.5081

Martínez-González, N., Atienza, F. L., Tomás, I., & Balaguer, I. (2021). Perceived coach-created motivational climates as predictors of athletes' goal reengagement: The mediational role of goal motives. *Frontiers in Psychology*, *12*, Article 740060. https://doi.org/10.3389/fpsyg.2021.740060

Mayer, Z., & Freund, A. M. (2022). Better off without? Benefits and costs of resolving goal conflict through goal shelving and goal disengagement. *Motivation and Emotion*, *46*(6), 790–805. https://doi.org/10.1007/s11031-022-09966-x

Messay, B., & Marsland, A. L. (2015). Goal adjustment ability predicts magnitude of emotional and physiological responses to an unsolvable anagram task. *Personality and Individual Differences*, *86*, 417–421. https://doi.org/10.1016/j.paid.2015.07.010

Miller, J. (2015). *Social problem-solving and suicidality* [Doctoral dissertation, University of Glasgow]. Enlighten Theses. https://eleanor.lib.gla.ac.uk/record=b3099863

Miyagawa, Y., Taniguchi, J., & Niiya, Y. (2018). Can self-compassion help people regulate unattained goals and emotional reactions toward setbacks? *Personality and Individual Differences*, *134*, 239–244. https://doi.org/10.1016/j.paid.2018.06.029

Munk, M. (2013). *Psychological correlates of primary versus secondary infertility*. Doctoral Thesis, Fairleigh Dickinson University. Proquest Dissertations and Theses. https://www.proquest.com/dissertations-theses/psychological-correlates-primary-versus-secondary/docview/1611898514/se-2?accountid=10382

Neely, M. E., Schallert, D. L., Mohammed, S. S., Roberts, R. M., & Chen, Y.-J. (2009). Self-kindness when facing stress: The role of self-compassion, goal regulation, and support in college students' well-being. *Motivation and Emotion*, *33*(1), 88–97. https://doi.org/10.1007/s11031-008-9119-8

Neff, K. D., & Faso, D. J. (2015). Self-compassion and well-being in parents of children with autism. *Mindfulness*, *6*(4), 938–947. https://doi.org/10.1007/s12671-014-0359-2

Neter, E., & Goren, S. (2017). Infertility centrality in the woman's identity and goal adjustment predict psychological adjustment among women in ongoing fertility treatments. *International Journal of Behavioral Medicine*, *24*(6), 880–892. https://doi.org/10.1007/s12529-017-9693-9

Neter, E., Litvak, A., & Miller, A. (2009). Goal disengagement and goal re-engagement among multiple sclerosis patients: Relationship to well-being and illness representation. *Psychology & Health*, *24*(2), 175–186. https://doi.org/10.1080/08870440701668665

Nicholls, A. R., Levy, A. R., Carson, F., Thompson, M. A., & Perry, J. L. (2016). The applicability of self-regulation theories in sport: Goal adjustment capacities, stress appraisals, coping, and well-being among athletes. *Psychology of Sport and Exercise*, *27*, 47–55. https://doi.org/10.1016/j.psychsport.2016.07.011

North, R. J., Holahan, C. J., Carlson, C. L., & Pahl, S. A. (2014). From failure to flourishing: The roles of acceptance and goal reengagement. *Journal of Adult Development*, *21*(4), 239–250. https://doi.org/10.1007/s10804-014-9195-9

Ntoumanis, N., Healy, L. C., Sedikides, C., Smith, A. L., & Duda, J. L. (2014). Self-regulatory responses to unattainable goals: The role of goal motives. *Self and Identity*, *13*(5), 594–612. https://doi.org/10.1080/15298868.2014.889033

O'Connor, R. C., & Forgan, G. (2007). Suicidal thinking and perfectionism: The role of goal adjustment and Behavioral Inhibition/Activation Systems (BIS/BAS). *Journal of Rational-Emotive & Cognitive-Behavior Therapy*, *25*(4), 321–341. https://doi.org/10.1007/s10942-007-0057-2

O'Connor, R. C., Fraser, L., Whyte, M.-C., MacHale, S., & Masterton, G. (2009). Self-regulation of unattainable goals in suicide attempters: The relationship between goal disengagement,

goal reengagement and suicidal ideation. *Behaviour Research and Therapy*, *47*(2), 164–169. https://doi.org/10.1016/j.brat.2008.11.001

O'Connor, R. C., O'Carroll, R. E., Ryan, C., & Smyth, R. (2012). Self-regulation of unattainable goals in suicide attempters: A two year prospective study. *Journal of Affective Disorders*, *142*(1–3), 248–255. https://doi.org/10.1016/j.jad.2012.04.035

O'Dea, C. (2014). *Goal motivation and the self-regulation of goals in depression*. Doctoral Thesis, University of Liverpool. University of Liverpool Repository. https://livrepository.liverpool.ac.uk/2009022/

Offerman, M. P. J., Schroevers, M. J., van der Velden, L.-A., de Boer, M. F., & Pruyn, J. F. A. (2010). Goal processes & self-efficacy related to psychological distress in head & neck cancer patients and their partners. *European Journal of Oncology Nursing*, *14*(3), 231–237. https://doi.org/10.1016/j.ejon.2010.01.022

Pat, L. Y. C., & Lau, B. H. P. (2023). Goal pursuing styles and the emergence of post-traumatic growth: a study with Hong Kong young adults under the 'double hit'of social movement and COVID. *Asia Pacific Journal of Counselling and Psychotherapy*, *14*(2), 112-127. https://doi.org/10.1080/21507686.2023.2278033

Patel, P. C., & Thatcher, S. M. B. (2014). Sticking it out: Individual attributes and persistence in self-employment. *Journal of Management*, *40*(7), 1932–1979. https://doi.org/10.1177/0149206312446643

Poderico, C., Ruggiero, G., Iachini, T., & Iavarone, A. (2006). Coping strategies and cognitive functioning in elderly people from a rural community in Italy. *Psychological Reports*, *98*(1), 159–168. https://doi.org/10.2466/pr0.98.1.159-168

Praskova, A., Creed, P. A., & Hood, M. (2013). Facilitating engagement in new career goals: The moderating effects of personal resources and career actions. *International Journal for Educational and Vocational Guidance*, *13*(2), 115–134. https://doi.org/10.1007/s10775-013-9242-2

Praskova, A., & McPeake, L. (2021). Career goal discrepancy, career distress, and goal adjustment: Testing a dual moderated process model in young adults. *Journal of Career Assessment*, *30*(4), 615–634. https://doi.org/10.1177/10690727211063372

Preiser, S., Auth, A., & Buttkewitz, S. (2005). Bewältigung von Lebensenttäuschungen-Innere und äußere Ressourcen. *Zeitschrift für Psychologie/Journal of Psychology*, *213*(1), 34-43. https://doi.org/10.1026/0044-3409.213.1.34

Price-Lee, J. (2024). *Finding the dream job or following another dream: Work volition and goal reengagement as predictors of work-filfillment, wellbeing and calling*. Doctoral Thesis, The University of South Dakota. https://red.library.usd.edu/diss-thesis/273

Raja, U., Naseer, S., & Khan, S. (2025). The pitfalls of career plateau: unravelling the mechanisms and conditions leading to adverse career outcomes. *International Journal of Organizational Analysis*, (ahead-of-print). https://doi.org/10.1108/IJOA-07-2024-4688

Ramírez-Maestre, C., Esteve, R., López-Martínez, A. E., Serrano-Ibáñez, E. R., Ruiz-Párraga, G. T., & Peters, M. (2019). Goal adjustment and well-being: The role of optimism in patients with chronic pain. *Annals of Behavioral Medicine*, *53*(7), 597–607. https://doi.org/10.1093/abm/kay070

Riddell, H., Sedikides, C., Gucciardi, D. F., Jackson, B., Thøgersen-Ntoumani, C., & Ntoumanis, N. (2022). Goal motives and mental contrasting with implementation intentions facilitate strategic goal persistence and disengagement. *Journal of Applied Social Psychology*, *52*(11), 1094–1116. https://doi.org/10.1111/jasp.12915

Riddell, H., Sedikides, C., Gucciardi, D. F., Jackson, B., Thøgersen-Ntoumani, C., & Ntoumanis, N. (2023). Motives and mental contrasting with implementation intentions predict progress and management of goals in parents. *Motivation Science*, *9*(2), 144–155. https://doi.org/10.1037/mot0000290

Riddell, H., Sedikides, C., Gucciardi, D. F., Sezer, B., Jackson, B., Thøgersen-Ntoumani, C., & Ntoumanis, N. (2024). Goal motives, mental contrasting with implementation intentions, and the self-regulation of saving goals: A longitudinal investigation. *Motivation Science, 10*(1), 28–39. https://doi.org/10.1037/mot0000311

Rider Mundey, K., Nicholas, D., Kruczek, T., Tschopp, M., & Bolin, J. (2019). Posttraumatic growth following cancer: The influence of emotional intelligence, management of intrusive rumination, and goal disengagement as mediated by deliberate rumination. *Journal of Psychosocial Oncology*, *37*(4), 456–477. https://doi.org/10.1080/07347332.2018.1514449

Rivers, S. (2014). *Optimism and mastery as psychosocial outcomes in women with primary ovarian insufficiency.* Doctoral Thesis, Walden University. Proquest Dissertations and Theses. https://www.proquest.com/dissertations-theses/optimism-mastery-as-psychosocial-outcomes-women/docview/1512435775/se-2?accountid=10382

Robertson, A, P. (2025). *Social support and adjusting major life goals: Potential predictors of prolonged grief disorder.* Doctoral Thesis, Alliant International University.

Rühs, F., Greve, W., & Kappes, C. (2017). Coping with criminal victimization and fear of crime: The protective role of accommodative self-regulation. *Legal and Criminological Psychology, 22*(2), 359–377. https://doi.org/10.1111/lcrp.12106

Sahdra, B. K., Ciarrochi, J., Basarkod, G., Dicke, T., Guo, J., Parker, P. D., & Marsh, H. W. (2022). High school students' tenacity and flexibility in goal pursuit linked to life satisfaction and achievement on competencies tests. *Journal of Educational Psychology, 114*(3), 622–636. https://doi.org/10.1037/edu0000667

Salimi, S., Nilforooshan, P., & Sadeghi, A. (2023). Towards career satisfaction by career adaptation model among individuals with visual impairment. *Journal of Career Development, 50*(1), 200–215. https://doi.org/10.1177/08948453221084138

Sasiela, W. J. (2020). *Depression and goal disengagement: The mediating role of goal attainability perceptions.* Master's Thesis, University of North Carolina at Greensboro. Proquest Dissertations and Theses Global. https://www.proquest.com/dissertations-theses/depression-goal-disengagement-mediating-role/docview/2429005274/se-2?accountid=10382

Sasiela, J. (2023). *The benefits of giving up: Clarifying how inabilities to disengage relate to depression*. Doctoral Thesis, University of North Carolina Greensboro. https://libres.uncg.edu/ir/uncg/f/Sasiela_uncg_0154D_13598.pdf

Schmitz, U., Saile, H., & Nilges, P. (1996). Coping with chronic pain: Flexible goal adjustment as an interactive buffer against pain-related distress. *Pain, 67*(1), 41–51. https://doi.org/10.1016/0304-3959(96)03108-9

Schroevers, M. J., Kraaij, V., & Garnefski, N. (2011). Cancer patients' experience of positive and negative changes due to the illness: Relationships with psychological well-being, coping, and

goal reengagement: Goal reengagement, coping, well-being, and changes in cancer patients. *Psycho-Oncology, 20*(2), 165–172. https://doi.org/10.1002/pon.1718

Schroevers, M., Kraaij, V., & Garnefski, N. (2008). How do cancer patients manage unattainable personal goals and regulate their emotions/. *British Journal of Health Psychology, 13*(3), 551–562. https://doi.org/10.1348/135910707X241497

Schüz, B., Wurm, S., Warner, L. M., & Tesch-Römer, C. (2009). Health and subjective well-being in later adulthood: Different health states-different needs. *Applied Psychology: Health and Well-Being, 1*(1), 23–45. https://doi.org/10.1111/j.1745-7254.2007.00657.x-i1

Shane, J. (2014). *The road taken: Social mobility in the transition to adulthood*. Doctoral Thesis, University of California Irvine. Proquest Dissertations and Theses Global. https://www.proquest.com/dissertations-theses/road-taken-social-mobility-transition-adulthood/docview/1648430712/se-2?accountid=10382

Shane, J., & Heckhausen, J. (2013). University students' causal conceptions about social mobility: Diverging pathways for believers in personal merit and luck. *Journal of Vocational Behavior, 82*(1), 10–19. https://doi.org/10.1016/j.jvb.2012.08.003

Shipley, C. (2019). *The relationship between self-compassion and eating behaviour.* Doctoral Thesis, University of Liverpool. The University of Liverpool Repository. https://livrepository.liverpool.ac.uk/id/eprint/3059065

Siltanen, S., Rantanen, T., Portegijs, E., Tourunen, A., Poranen-Clark, T., Eronen, J., & Saajanaho, M. (2019). Association of tenacious goal pursuit and flexible goal adjustment with out-of-home mobility among community-dwelling older people. *Aging Clinical and Experimental Research, 31*(9), 1249–1256. https://doi.org/10.1007/s40520-018-1074-y

Sisi, Q., Jinfeng, Z., Lili, W., & Jianxin, Z. (2021). Attachment styles, self-esteem, flexible goal adjustment, and intimate relationship satisfaction in women: A moderated mediation model. *The Journal of Psychology, 155*(4), 426–440. https://doi.org/10.1080/00223980.2021.1896463

Skhirtladze, N., Luyckx, K., & Schwartz, S. J. (2021). Longitudinal associations between Identity processes and goal engagement and disengagement: Directionality of effects and correlated change. *Social Development, 30*(3), 833–849. https://doi.org/10.1111/sode.12502

Smagula, S. F., Faulkner, K., Scheier, M. F., Tindle, H. A., Cauley, J. A., & for the Osteoporotic Fractures in Men (MrOS) Study Group. (2016). Testing the independence of multiple personality factors in relation to health among community-dwelling older men. *Journal of Aging and Health, 28*(4), 571–586. https://doi.org/10.1177/0898264315597649

Smith, A. L., & Ntoumanis, N. (2014). An examination of goal motives and athletes' self-regulatory responses to unattainable goals. *International Journal of Sport Psychology, 45*(6), 538–558. https://doi.org/10.7352/IJSP2014.45.538

Stark, H. J. (2000). *Adaptability, social activity, and well-being among older adults.* Doctoral Thesis, California School of Professional Psychology. Proquest Dissertations and Theses Global. https://www.proquest.com/dissertations-theses/adaptability-social-activity-well-being-among/docview/304610452/se-2?accountid=10382

Sugimoto, K. (2025). Grit, coping strategies for difficult goals, and well-being. *The Japanese Journal of Educational Psychology, 73*(1), 1–13. https://doi.org/10.5926/jjep.73.1

Swindells, T., Iddon, J., & Dickson, J. (2023). The role of adaptive goal processes in mental wellbeing in chronic pain. *International Journal Of Environmental Research And Public Health*, *20*(2), Article 1278. https://doi.org/10.3390/ijerph20021278

Thompson, E. H., Woodward, J. T., & Stanton, A. L. (2011). Moving forward during major goal blockage: Situational goal adjustment in women facing infertility. *Journal of Behavioral Medicine*, *34*(4), 275–287. https://doi.org/10.1007/s10865-010-9309-1

Thompson, E., Stanton, A. L., & Bower, J. E. (2013). Situational and dispositional goal adjustment in the context of metastatic cancer: Goal adjustment and metastatic cancer. *Journal of Personality*, *81*(5), 441–451. https://doi.org/10.1111/jopy.12025

Thomsen, T., & Greve, W. (2013). Accommodative coping in early adolescence: An investigation of possible developmental components. *Journal of Adolescence*, *36*(5), 971–981. https://doi.org/10.1016/j.adolescence.2013.08.003

Thomsen, T., Kappes, C., Schwerdt, L., Sander, J., & Poller, C. (2017). Modelling goal adjustment in social relationships: Two experimental studies with children and adults. *British Journal of Developmental Psychology*, *35*(2), 267–287. https://doi.org/10.1111/bjdp.12162

Tobin, S. J., & Raymundo, M. M. (2010). Causal uncertainty and psychological well-being: The moderating role of accommodation (secondary control). *Personality and Social Psychology Bulletin*, *36*(3), 371–383. https://doi.org/10.1177/0146167209359701

Tolentino, L. R. (2015). *Bending with the wind: An integrative process model of career adaptation.* Doctoral Thesis, Australian National University. Open Research Library. doi.org/10.25911/5d78d51163c81

Tolentino, L. R., Garcia, P. R. J. M., Restubog, S. L. D., Bordia, P., & Tang, R. L. (2013). Validation of the Career Adapt-Abilities Scale and an examination of a model of career adaptation in the Philippine context. *Journal of Vocational Behavior*, *83*(3), 410-418. https://doi.org/10.1016/j.jvb.2013.06.013

Tomasik, M. J., Silbereisen, R. K., & Heckhausen, J. (2010). Is it adaptive to disengage from demands of social change? Adjustment to developmental barriers in opportunity-deprived regions. *Motivation and Emotion*, *34*(4), 384–398. https://doi.org/10.1007/s11031-010-9177-6

Tovel, H., & Carmel, S. (2014). Maintaining successful aging: The role of coping patterns and resources. *Journal of Happiness Studies*, *15*(2), 255–270. https://doi.org/10.1007/s10902-013-9420-4

Toyama, M. (2024). Is grit persistence adaptive? Goal pursuit behavior when faced with a difficult goal. *Personality and individual differences, 223*, 112610. https://doi.org/10.1016/j.paid.2024.112610

Urbanaviciute, I., Kairys, A., Paradnikė, K., & Pociute, B. (2019). Capturing serendipity in careers: An evaluation of the planned happenstance career inventory with Lithuanian undergraduates. *Journal of Career Development*, *46*(2), 157–170. https://doi.org/10.1177/0894845317731158

Van Bost, G., Van Damme, S., & Crombez, G. (2020). Goal reengagement is related to mental well-being, life satisfaction and acceptance in people with an acquired brain injury. *Neuropsychological Rehabilitation*, *30*(9), 1814–1828. https://doi.org/10.1080/09602011.2019.1608265

Van Bost, G., Van Damme, S., & Crombez, G. (2022). Goal adjustment and well-being after an acquired brain injury: The role of cognitive flexibility and personality traits. *PeerJ, 10*, Article e13531. https://doi.org/10.7717/peerj.13531

Van Damme, S., De Waegeneer, A., & Debruyne, J. (2016). Do flexible goal adjustment and acceptance help preserve quality of life in patients with multiple sclerosis? *International Journal of Behavioral Medicine*, *23*(3), 333–339. https://doi.org/10.1007/s12529-015-9519-6

Van Damme, S., Kindt, S., Crombez, G., Goubert, L., & Debruyne, J. (2019). The relation between goal adjustment, goal disturbance, and mental well-being among persons with multiple sclerosis. *Psychology & Health*, *34*(6), 645–660. https://doi.org/10.1080/08870446.2018.1556272

Van Diemen, T., Van Nes, I. J. W., Geertzen, J. H. B., & Post, M. W. M. (2018). Coping fexibility as predictor of distress in persons with spinal cord injury. *Archives of Physical Medicine and Rehabilitation*, *99*(10), 2015–2021. https://doi.org/10.1016/j.apmr.2018.05.032

Van Lankveld, W., van Diemen, T., & van Nes, I. J. (2011). Coping with spinal cord injury: Tenacious goal pursuit and flexible goal adjustment. *Journal of Rehabilitation Medicine, 43*(10), 923-929. https://dx.doi.org/10.2340/16501977-0870

Van Luenen, S., Kraaij, V., Spinhoven, P., Wilderjans, T. F., & Garnefski, N. (2019). Exploring mediators of a guided web-based self-help intervention for people with HIV and depressive symptoms: Randomized controlled trial. *JMIR Mental Health*, *6*(8), Article e12711. https://doi.org/10.2196/12711

Vann, R. J., Rosa, J. A., & McCrea, S. M. (2018). When consumers struggle: Action crisis and its effects on problematic goal pursuit. *Psychology & Marketing*, *35*(9), 696–709. https://doi.org/10.1002/mar.21116

Verschuren, A., & Douilliez, C. (2024). Goal disengagement and goal reengagement: Associations with depression, anxiety, and satisfaction with life. *Canadian Journal of Behavioural Science / Revue Canadienne Des Sciences Du Comportement, 6*(2), 93–101. https://doi.org/10.1037/cbs0000360

Visser, M., Aben, L., Heijenbrok-Kal, M., Busschbach, J., & Ribbers, G. (2014). The relative effect of coping strategy and depression on health-related quality of life in patients in the

chronic phase after stroke. *Journal of Rehabilitation Medicine*, *46*(6), 514–519. https://doi.org/10.2340/16501977-1803

Vohs, K. D., Park, J. K., & Schmeichel, B. J. (2013). Self-affirmation can enable goal disengagement. *Journal of Personality and Social Psychology*, *104*(1), 14–27. https://doi.org/10.1037/a0030478

Von Blanckenburg, P., Seifart, U., Conrad, N., Exner, C., Rief, W., & Nestoriuc, Y. (2014). Quality of life in cancer rehabilitation: The role of life goal adjustment. *Psycho-Oncology*, *23*(10), 1149–1156. https://doi.org/10.1002/pon.3538

Von Keyserlingk, L., Rubach, C., Lee, H., Eccles, J., & Heckhausen, J. (2022). College Students' motivational beliefs and use of goal-oriented control strategies: Integrating two theories of motivated behavior. *Motivation And Emotion*, *46*(5), 601–620. https://doi.org/10.1007/s11031-022-09957-y

Wen, B., Zhang, M., Zhang, L., Zhou, Y., & Xu, L. (2023). How over-parenting impedes individual career exploration: A goal disengagement perspective. *BMC Psychology*, *11*, Article 109. https://doi.org/10.1186/s40359-023-01163-w

Widyowati, A., Hood, M., Duffy, A., & Creed, P. (2024). Negative Career Goal Discrepancy and Goal Adjustment in Young Adults: The Underlying Mechanism of Negative Emotions. *Journal of Career Development*, *51*(2), 234-253. https://doi.org/10.1177/08948453241235406

Windsor, T. D., & Wilton-Harding, B. (2025). Examining goal re-engagement as a mediator of longitudinal associations between awareness of age-related change and vitality in midlife and older adulthood. *International Journal of Behavioral Development*, *49*(2), 155-166. https://doi.org/10.1177/01650254241298896

Wrosch, C., & Miller, G. E. (2009). Depressive symptoms can be useful: Self-regulatory and emotional benefits of dysphoric mood in adolescence. *Journal of Personality and Social Psychology*, *96*(6), 1181–1190. https://doi.org/10.1037/a0015172

Wrosch, C., Miller, G. E., Scheier, M. F., & de Pontet, S. B. (2007). Giving up on unattainable goals: Benefits for health? *Personality and Social Psychology Bulletin*, *33*(2), 251–265. https://doi.org/10.1177/0146167206294905

Wrosch, C., Rueggeberg, R., & Hoppmann, C. A. (2013). Satisfaction with social support in older adulthood: The influence of social support changes and goal adjustment capacities. *Psychology and Aging, 28*(3), 875–885. https://doi.org/10.1037/a0032730

Wrosch, C., & Sabiston, C. M. (2013). Goal adjustment, physical and sedentary activity, and well-being and health among breast cancer survivors: Goal adjustment, lifestyle activity, and well-being and health. *Psycho-Oncology, 22*(3), 581–589. https://doi.org/10.1002/pon.3037

Wrosch, C., Scheier, M. F., Miller, G. E., Schulz, R., & Carver, C. S. (2003). Adaptive self-regulation of unattainable goals: Goal disengagement, goal reengagement, and subjective well-being. *Personality and Social Psychology Bulletin, 29*(12), 1494–1508. https://doi.org/10.1177/0146167203256921

Yau, P. S., Cho, Y., Kay, J., & Heckhausen, J. (2022a). The effect of motive-goal congruence on adolescents' academic goal engagement and disengagement. *Motivation and Emotion, 46*(4), 447–460. https://doi.org/10.1007/s11031-022-09946-1

Yau, P. S., Cho, Y., Shane, J., Kay, J., & Heckhausen, J. (2022b). Parenting and adolescents' academic achievement: The mediating role of goal engagement and disengagement. *Journal of Child and Family Studies, 31*(4), 897–909. https://doi.org/10.1007/s10826-021-02007-0

Zhang, J. (2020). Tenacious goal pursuit, flexible goal adjustment, and life satisfaction among Chinese older adult couples. *Research on Aging, 42*(1), 13–22. https://doi.org/10.1177/0164027519876125

Zhang, J., Nancy Xiaonan, Y., Zhang, J., & Zhou, M. (2018). Age stereotypes, flexible goal adjustment, and well-being among Chinese older adults. *Psychology, Health & Medicine, 23*(2), 210–215. https://doi.org/10.1080/13548506.2017.1344253

Zhu, L., Ranchor, A. V., van der Lee, M., Garssen, B., Sanderman, R., & Schroevers, M. J. (2015). The role of goal adjustment in symptoms of depression, anxiety and fatigue in cancer patients receiving psychosocial care: A longitudinal study. *Psychology & Health, 30*(3), 268–283. https://doi.org/10.1080/08870446.2014.969263
