# Supplementary material for: A meta-analytic review and conceptual model of the antecedents and outcomes of goal adjustment in response to striving difficulties
Source: Nat Hum Behav. 2025 Nov 13;10(2):317–32. doi: 10.1038/s41562-025-02312-4 (PMC12932095; doi:10.1038/s41562-025-02312-4)
Supplement: Supplementary file 2 — Reporting Summary [file 41562_2025_2312_MOESM2_ESM.pdf]

nature portfolio

Corresponding author(s): *Double-anonymous peer review submissions: write DAPR and your manuscript number here instead of author names.*

Last updated by author(s): *YYYY-MM-DD*

Reporting Summary

Nature Portfolio wishes to improve the reproducibility of the work that we publish. This form provides structure for consistency and transparency in reporting. For further information on Nature Portfolio policies, see our Editorial Policies and the Editorial Policy Checklist.

Statistics

For all statistical analyses, confirm that the following items are present in the figure legend, table legend, main text, or Methods section.

n/a	Confirmed	
☐	☒	The exact sample size (n) for each experimental group/condition, given as a discrete number and unit of measurement
☒	☐	A statement on whether measurements were taken from distinct samples or whether the same sample was measured repeatedly
☐	☒	The statistical test(s) used AND whether they are one- or two-sided<br>*Only common tests should be described solely by name; describe more complex techniques in the Methods section.*
☐	☒	A description of all covariates tested
☐	☒	A description of any assumptions or corrections, such as tests of normality and adjustment for multiple comparisons
☐	☒	A full description of the statistical parameters including central tendency (e.g. means) or other basic estimates (e.g. regression coefficient) AND variation (e.g. standard deviation) or associated estimates of uncertainty (e.g. confidence intervals)
☐	☒	For null hypothesis testing, the test statistic (e.g. F, t, r) with confidence intervals, effect sizes, degrees of freedom and P value noted<br>*Give P values as exact values whenever suitable.*
☒	☐	For Bayesian analysis, information on the choice of priors and Markov chain Monte Carlo settings
☐	☒	For hierarchical and complex designs, identification of the appropriate level for tests and full reporting of outcomes
☐	☒	Estimates of effect sizes (e.g. Cohen's d, Pearson's r), indicating how they were calculated

Our web collection on statistics for biologists contains articles on many of the points above.

Software and code

Policy information about availability of computer code

Data collection	Endnote was use to collate articles identified through the systematic search
Data analysis	All analysis was We conducted all data analyses in R (Version 4.5; R Core Team, 2022) using the metafor (Veichtbauer, 2010) and metaviz packages (Kossmeier et al., 2020). All analysis code has been published on the project's OSF page (https://osf.io/6qft4/).

For manuscripts utilizing custom algorithms or software that are central to the research but not yet described in published literature, software must be made available to editors and reviewers. We strongly encourage code deposition in a community repository (e.g. GitHub). See the Nature Portfolio guidelines for submitting code & software for further information.

Data

Policy information about availability of data

All manuscripts must include a data availability statement. This statement should provide the following information, where applicable:

- Accession codes, unique identifiers, or web links for publicly available datasets
- A description of any restrictions on data availability
- For clinical datasets or third party data, please ensure that the statement adheres to our policy

All data and analysis scripts for this project have been made publicly accessible via the Open Science Framework (https://osf.io/6qft4/).

Research involving human participants, their data, or biological material

Policy information about studies with human participants or human data. See also policy information about sex, gender (identity/presentation), and sexual orientation and race, ethnicity and racism.

Reporting on sex and gender	We included participant gender distribution (% female participants) as a moderator in our analyses. Most included studies did not explicitly describe biological sex of participants, therefore we were unable to conduct analyses pertaining to biological sex and operate under the assumption that gender reported in the included studies reflects identified gender not biological sex.
Reporting on race, ethnicity, or other socially relevant groupings	We analyzed the country in which study samples were drawn from as a moderator, but did not include further information about the explicit race or ethnicity of participants within these samples, as this information was not clear in from the majority of included manuscripts.
Population characteristics	In addition to the above mentioned population characteristics, we included participant age and participant subsample as moderators in our analyses
Recruitment	N/A
Ethics oversight	N/A

Note that full information on the approval of the study protocol must also be provided in the manuscript.

Field-specific reporting

Please select the one below that is the best fit for your research. If you are not sure, read the appropriate sections before making your selection.

☐ Life sciences ☒ Behavioural & social sciences ☐ Ecological, evolutionary & environmental sciences

For a reference copy of the document with all sections, see nature.com/documents/nr-reporting-summary-flat.pdf

Behavioural & social sciences study design

All studies must disclose on these points even when the disclosure is negative.

Study description	Meta-analysis of qualitative studies
Research sample	This study was a meta-analysis it synthesised effect sizes from multiple samples. Given we extracted information from 281 samples, it would be impractical to provide full details here. We provide information about individual samples included in the meta-analysis in Supplementary Table ST5
Sampling strategy	We conducted a systematic search of the following electronic databases: Web of Science, Scopus, PsycInfo, Business Source Ultimate, Proquest Dissertations and Theses Global, Medline. Studies were eligible if they measured goal adjustment in any form (e.g., self-reported adjustment capacity, objectively assessed behavioural adjustment) and either one or all of the following: (i) antecedents predicting goal adjustment; (ii) goal-related outcomes; (iii) functional outcomes; (iv) wellbeing outcomes.
Data collection	We extracted effect sizes (r) from articles that met our inclusion criteria and synthesised pooled effect size estimates using three-level meta-regression.
Timing	The search was initially conducted on May 25, 2022, updated on July 15, 2023 and updated again on May 22, 2025
Data exclusions	no data were excluded
Non-participation	N/A
Randomization	N/A

Reporting for specific materials, systems and methods

We require information from authors about some types of materials, experimental systems and methods used in many studies. Here, indicate whether each material, system or method listed is relevant to your study. If you are not sure if a list item applies to your research, read the appropriate section before selecting a response.

Materials & experimental systems

n/a	Involved in the study
☒	☐ Antibodies
☒	☐ Eukaryotic cell lines
☒	☐ Palaeontology and archaeology
☒	☐ Animals and other organisms
☒	☐ Clinical data
☒	☐ Dual use research of concern
☒	☐ Plants

Methods

n/a	Involved in the study
☒	☐ ChIP-seq
☒	☐ Flow cytometry
☒	☐ MRI-based neuroimaging

Plants

Seed stocks	*Report on the source of all seed stocks or other plant material used. If applicable, state the seed stock centre and catalogue number. If plant specimens were collected from the field, describe the collection location, date and sampling procedures.*
Novel plant genotypes	*Describe the methods by which all novel plant genotypes were produced. This includes those generated by transgenic approaches, gene editing, chemical/radiation-based mutagenesis and hybridization. For transgenic lines, describe the transformation method, the number of independent lines analyzed and the generation upon which experiments were performed. For gene-edited lines, describe the editor used, the endogenous sequence targeted for editing, the targeting guide RNA sequence (if applicable) and how the editor was applied.*
Authentication	*Describe any authentication procedures for each seed stock used or novel genotype generated. Describe any experiments used to assess the effect of a mutation and, where applicable, how potential secondary effects (e.g. second site T-DNA insertions, mosiacism, off-target gene editing) were examined.*
